# The Use of Evidence to Design an Essential Package of Health Services in Pakistan: A Review and Analysis of Prioritisation Decisions at Different Stages of the Appraisal Process

**DOI:** 10.34172/ijhpm.2024.8043

**Published:** 2024-03-09

**Authors:** Sergio Torres-Rueda, Anna Vassall, Raza Zaidi, Nichola Kitson, Muhammad Khalid, Wahaj Zulfiqar, Maarten Jansen, Wajeeha Raza, Maryam Huda, Frank Sandmann, Rob Baltussen, Sameen Siddiqi, Ala Alwan

**Affiliations:** ^1^Department of Global Health & Development, London School of Hygiene and Tropical Medicine, London, UK.; ^2^Ministry of National Health Services, Regulations and Coordination, Islamabad, Pakistan.; ^3^Department of Health Evidence, Radboud Institute of Health Sciences, Radboud University Medical Center, Nijmegen, The Netherlands.; ^4^Centre for Health Economics, University of York, York, UK.; ^5^Department of Community Health Sciences, Aga Khan University, Karachi, Pakistan.; ^6^Department of Infectious Disease Epidemiology, London School of Hygiene and Tropical Medicine, London, UK.; ^7^DCP3 Country Translation Project, London School of Hygiene and Tropical Medicine, London, UK.

**Keywords:** Health Benefit Packages, Essential Packages of Health Services, Pakistan, Priority Setting, Cost-Effectiveness, Decision Criteria

## Abstract

**Background:** Pakistan embarked on a process of designing an essential package of health services (EPHS) as a pathway towards universal health coverage (UHC). The EPHS design followed an evidence-informed deliberative process; evidence on 170 interventions was introduced along multiple stages of appraisal engaging different stakeholders tasked with prioritising interventions for inclusion. We report on the composition of the package at different stages, analyse trends of prioritised and deprioritised interventions and reflect on the trade-offs made.

**Methods:** Quantitative evidence on cost-effectiveness, budget impact, and avoidable burden of disease was presented to stakeholders in stages. We recorded which interventions were prioritised and deprioritised at each stage and carried out three analyses: (1) a review of total number of interventions prioritised at each stage, along with associated costs per capita and disability-adjusted life years (DALYs) averted, to understand changes in affordability and efficiency in the package, (2) an analysis of interventions broken down by decision criteria and intervention characteristics to analyse prioritisation trends across different stages, and (3) a description of the trajectory of interventions broken down by current coverage and cost-effectiveness.

**Results:** Value for money generally increased throughout the process, although not uniformly. Stakeholders largely prioritised interventions with low budget impact and those preventing a high burden of disease. Highly cost-effective interventions were also prioritised, but less consistently throughout the stages of the process. Interventions with high current coverage were overwhelmingly prioritised for inclusion.

**Conclusion:** Evidence-informed deliberative processes can produce actionable and affordable health benefit packages. While cost-effective interventions are generally preferred, other factors play a role and limit efficiency.

## Background

**Key Messages**

**Implications for policy makers**

- This study examines trends in the composition of the essential packages of health services (EPHS) during different stages of the priority setting process in Pakistan. We found that evidence-informed deliberative processes in low- and middle-income settings can effectively lead to the design of EPHS that are affordable and represent good value for money.
- Our analysis suggests that value for money for the EPHS generally increased throughout the process. Stakeholders prioritised interventions with low budget impact and those preventing a high burden of disease. Cost-effectiveness was prioritised but other criteria beyond overall efficiency were also important. Reflecting on what those values are in an explicit manner could improve transparency in the process.
- Interventions with high current coverage, regardless of cost-effectiveness, were overwhelmingly prioritised for inclusion despite a structured decision-making process and evidence availability. While this trade-off suggests a possible aversion to disinvestment, issues around intervention feasibility may also be important considerations for policy-makers.

**Implications for the public**

The process of designing essential packages of health services (EPHS) is used globally to best utilise available resources and maximise health. Assuming specified budgets, such priority setting exercises require trading off health interventions considered for inclusion in the EPHS. In this paper we study how different types of interventions were prioritised during the EPHS design process in Pakistan. We find that value for money of the EPHS increased throughout the process; stakeholders prioritised affordable interventions that addressed diseases with high levels of morbidity and mortality. However, stakeholders also prioritised interventions that were already implemented in Pakistan and that already had high levels of coverage, even when these represented low value for money. To ensure that an EPHS is acceptable and sustainable, it is important to understand what types of values are prioritised by stakeholders involved in designing the EPHS and reflect how they match those of the general population.

Establishing an essential package of health services (EHPS) is a critical part of the pathway towards universal health coverage (UHC).^1^ Several low- and middle-income countries (LMICs) have revised or developed an EPHS in recent years,^2,3^ some of which can be found in the public domain.^4-6^ While approaches vary, the process often involves the review of intervention-specific evidence across several decision criteria by groups of stakeholders in a sequenced manner.^7-9^ Pakistan embarked on such a process in 2019-2020, producing an EPHS focused on district-level services.

 Given the large numbers of potential health interventions, as well as fixed budgets and other constraints, EPHS design processes require prioritising interventions and considering trade-offs across several decision criteria (eg, cost-effectiveness or budget impact) and intervention characteristics (eg, delivery platform or target population). A body of evidence from high-income countries (HICs) has quantitatively explored the relative importance of decision criteria in incremental health technology assessment (HTA) outcomes, suggesting cost-effectiveness is a highly influential criterion,^10-18^ among several others.^11-14,16-18^ However, little is known about the influence of decision criteria and intervention characteristics in broader EPHS design processes generally, particularly in LMICs.

 Further, different decision criteria may be influential at different stages of the process. Designing an EPHS often involves stepwise deliberative approaches that engage different groups of stakeholders; packages are typically reviewed by technical experts and national and provincial actors within the health system and approved and adopted by decision-makers. While the importance of understanding the process outcomes is evident, analysing the trajectory of individual interventions appraised throughout the EPHS design process is also key as it reveals how evidence is appraised and what decision criteria and intervention characteristics are ultimately prioritised by different types of stakeholders.

 EPHS design may entail reducing or altogether suspending the public provision of certain existing services. Evidence from HICs suggests disinvestment decisions are comparatively rare^6^ and hindered by a number of barriers in policy and practice.^19-25^ Consequently, inefficient interventions may not be explicitly removed from an EPHS, further limiting the fiscal space available to introduce more cost-effective interventions.^26^ However, little is known on what factors facilitate or hinder disinvestment in LMICs.

 The research aims of the paper are to report on the composition of the Pakistan EPHS at each stage of the appraisal process and explore which decision criteria and intervention characteristics were valued as important. We reflect both on the outcomes of each appraisal stage and on how the design of the appraisal process may have influenced it. We do so through three analyses: (*i*) a review of the total number of interventions, costs, and health outcomes (ie, disability-adjusted life years, or “DALYs,” averted) per appraisal stage to understand EPHS optimisation throughout the process; (*ii*) an analysis of interventions prioritised, categorised by decision criteria ranking and intervention characteristics, to analyse prioritisation trends across different appraisal stages; and (*iii*) a description of the trajectory of interventions, broken down by cost-effectiveness and current coverage, to highlight patterns in investment and disinvestment decisions and key trade-offs.

## Methods

###  Study Context and Background

 Pakistan, a lower-middle income country, is the fifth most populous country in the world, with an estimated population surpassing 220 million inhabitants.^27^ Governance, including on health matters, is largely devolved to provincial authorities.^28^ Neonatal mortality is among the highest in the world and neonatal disorders account for 17% of annual deaths.^29^ Health spending is almost entirely financed domestically and largely through out-of-pocket expenditure (54%).^30^

 The Pakistan EPHS development process used the Disease Control Priorities 3’s (DCP3’s) essential universal health coverage (EUHC) model package of 218 interventions as a framework of reference. In April 2019, the Ministry of National Health Services, Regulations & Coordination (MNHSR&C) of Pakistan carried out a scoping review and consultations with provincial-level stakeholders and the Health Planning, System Strengthening & Information Analysis Unit, to compare the composition of the 218 EUHC interventions to existing services and discuss their relevance to the Pakistani context. An initial shortlist of 170 EUHC interventions was suggested for further assessment. An evidence-informed deliberative process was used to prioritise interventions with the aim of defining an actionable, publicly funded package within fiscal space.^31^ This paper is part of a five-paper series, which provides in-depth information of the Pakistani context, the EPHS decision-making processes, evidence used and outcomes.^32-35^

###  Evidence and Assessment

 Eight decision criteria for assessment were selected by the MNHSR&C: effectiveness, cost-effectiveness, budget impact, avoidable burden of disease, feasibility, equity, financial risk protection, and socio-economic impact.^33^ While formal techniques such as quantitative multi-criteria decision analysis aim to weigh decision criteria (vis-à-vis one another) explicitly,^34,36^ the EPHS process in Pakistan employed a mix of quantitative and qualitative approaches in interpreting intervention performance. Quantitative evidence was collected, collated and presented on cost-effectiveness, budget impact and burden of disease as described in other papers in this series,^34,35^ and in the General Appendix. Resource requirements, which can partially indicate intervention feasibility, were also defined and presented.^37^ Intervention-specific evidence was not available for the remaining four decision criteria.

###  Appraisal

**Figure 1:**
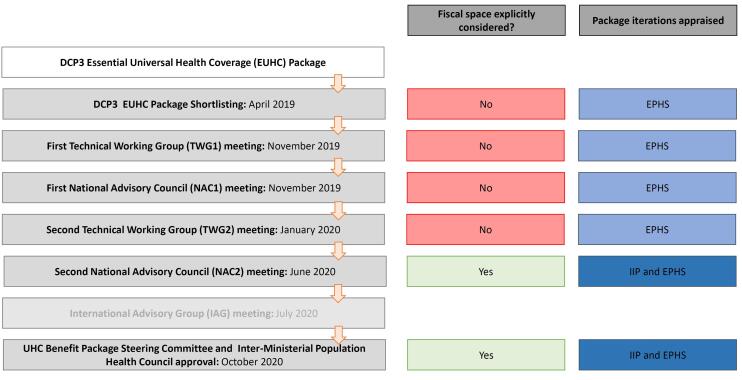
Timeline of Appraisal Process and Information on Budget Constraints, Intervention Levels Considered and Packages Reviewed Per Stage of The Process. Abbreviations: EPHS, essential package of health services; DCP3, Disease Control Priorities 3; IIP, immediate implementation package; UHC, universal health coverage.

 The appraisal process has been described in detail by Baltussen et al^33^ and Alwan et al^32^ and is explained in Figure 1. Evidence was reviewed and appraised by different stakeholders, in a sequential process. At each stage, a recommendation on whether to prioritise or deprioritise an intervention was agreed upon and documented. Recommendations at each stage were non-binding; a recommendation to prioritise or deprioritise an intervention at one stage could be reversed at a subsequent stage.

 Technical experts appraised evidence for all interventions in two technical working group meetings (TWG1 and TWG2) and proposed a list of prioritised interventions to the National Advisory Council (NAC), composed of stakeholders representing the MNHSR&C, societal interests, development partners and provincial representation, plus one representative from the TWG. Evidence on a subset of interventions was reviewed at an initial NAC meeting (NAC1). A second NAC meeting (NAC2) appraised all prioritised interventions within a given fiscal space, considering a range of scenarios to crystalise trade-offs. See the General Appendix for further details. Further, stakeholders involved in NAC2 agreed to proceed with the design of two packages, reflecting different time horizons and fiscal space challenges: a reduced immediate implementation package (IIP) to be rolled out over 2 years, and the full EPHS, to be implemented in stepwise manner over the following decade as health budgets improve.^32^ An International Advisory Group (IAG) provided further advice on intervention prioritisation. The packages suggested by NAC2, as well as IAG input, were considered by the UHC EPHS Steering Committee (UHC-EPHS SC) and the Inter-Ministerial Population Health Council (IMPHC), which reviewed and approved final iterations of both the IIP and full EPHS.

 Some countries, including Kazakhstan and Liberia, have used similar evidence-informed deliberative approaches for EPHS design while others, such as Iran, used this approach for disease-specific packages.^39^ A study comparing stepwise approaches in six countries — Afghanistan, Ethiopia, Pakistan, Somalia, Sudan, and Zanzibar (Tanzania) — found that all countries set up Advisory Committee groups.^38^ In all but one (Somalia) the process was supported by technical subcommittees providing specific disease programme information. While the decision criteria varied by country, all six countries assessed and appraised all interventions considered. Qualitative approaches to prioritisation were used in most countries although in Sudan quantitative approaches to scoring and weighting also played a role. Advisory Committees in all countries arrived at a final package through consensus (as opposed to a majority vote).

###  Analysis of Costs, Outcomes and Prioritised Decision Criteria and Intervention Characteristics During the Process

 We trace the trajectory of interventions throughout the process and carry out three types of analysis. For each of the three analyses, we present two sets of results to account for the process leading to both the full EPHS and the IIP. For analytical purposes, we do not include the results of NAC1 as it did not consider the entire package or prioritise among all 170 interventions. We also present the outcomes of TWG1 and TWG2 in a combined manner (henceforth “combined TWG”). Three analyses were conducted to understand changes in prioritisation throughout EPHS design process.

*( i ) Size, costs, and effects at different stages of the appraisal process:* The first analysis is an overall review of the total number of interventions prioritised in each stage of the appraisal process, along with the associated total costs per capita and total DALYs averted of prioritised interventions, as well as a calculation of total DALYs averted per dollar per capita spent. It is important to note that the definition of a “prioritised intervention” varies between appraisal stages and reflects the aim of each stage. To carry out this analysis we reviewed records compiled at each stage of the decision-making process (eg, logs detailing decisions at each stage) in order to produce a dataset of interventions included and excluded. This dataset was combined with data on budget impact, intervention effectiveness and burden of disease.^34,35,40^

*(ii) Composition of the package throughout the appraisal process*: The second analysis traces the trajectory of prioritised interventions by decision criteria and intervention characteristics. These criteria and characteristics were chosen by reviewing the literature on factors that influence priority setting decisions. We arrived at 10 criteria and characteristics that were highlighted in the literature, were present in the decision-making process in Pakistan, and were measurable in the context of our study.

 Firstly, we grouped interventions together by how they fared on the three decision criteria which were assessed quantitatively, as per evidence presented during the appraisal: (1) cost-effectiveness, (2) budget impact, (3) burden of preventable disease, as well as by (4) quality of available evidence on cost-effectiveness (incremental cost-effectiveness ratio quality, “ICER quality”). We also broke down interventions together by stated characteristics: (5) delivery platform, and (6) intervention cluster. Further, as part of our analysis, we grouped interventions by “implied” characteristics, which appraisers are likely to have identified: (7) intervention purpose, and whether the intervention could be defined as (8) addressing the health needs of a vulnerable population (here defined as involving reproductive, maternal, neonatal, child and adolescent health, or “RMNCAH,” as agreed by the NAC due to equity implications), or whether (9) the “rule of rescue” (defined as the imperative to rescue identifiable individuals facing avoidable death)^41^ was expected to apply. Lastly, we also examined (10) current coverage, defined as an estimate percentage of the target population receiving the intervention at the time of the deliberations. We compiled a spreadsheet which listed how each intervention scored (or was categorised) in each decision criteria or intervention characteristic. This dataset was combined with the data used for analysis (*i*) in order to understand which interventions, exhibiting particular decision criteria scores or characteristics, were prioritised at different points in the process.

*(iii) Distribution of interventions by current coverage and cost-effectiveness*: The third analysis describes the trajectory of interventions broken down by current coverage combined with cost-effectiveness. We categorised data on current intervention coverage for 2019 into four categories: high, medium, low and no coverage. Intervention cost-effectiveness was categorised as high, medium, and low cost-effectiveness and no cost-effectiveness evidence. We combined the two criteria to create sixteen joint indicators to describe each intervention (eg, high coverage and high cost-effectiveness, high coverage and medium cost-effectiveness, etc). We then combined this dataset with the data used for analysis (*i*) to understand which types of interventions were included and excluded vis-à-vis their cost-effectiveness and coverage, simultaneously.

 Further details on the methods used and on the decision criteria analysed can be found in the General Appendix. Supplementary files 1-3 contain the values used for each intervention per decision criteria or intervention characteristics and information on intervention inclusion and exclusion at each stage.

## Results

**Figure 2:**
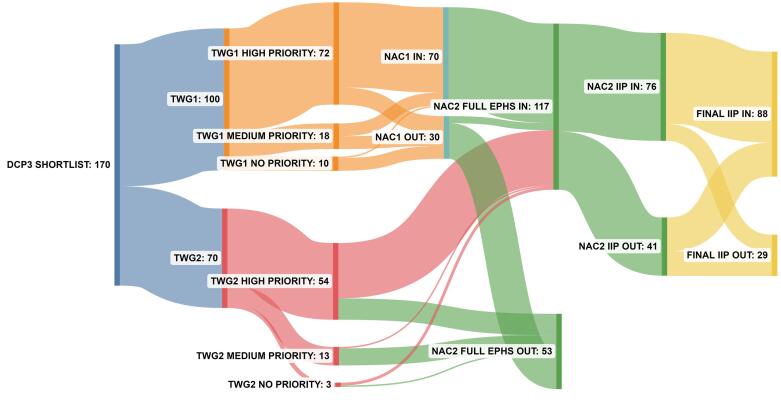
Trajectory of Interventions Throughout the Stages of the Full EPHS and IIP Appraisal Process Specifying Number of Interventions Included (IN) and Excluded (OUT) at Each Stage. Abbreviations: EPHS, essential package of health services; DCP3, Disease Control Priorities 3; IIP, immediate implementation package; NAC, National Advisory Council; TWG, technical working group.

Figure 2 shows the number of interventions prioritised at each stage of the appraisal process, concluding in the full EPHS, composed of 117 interventions and an IIP with 88 interventions. Details on the interventions included in the two final packages are presented in full in Alwan et al.^32^

###  Size, Costs, and Effects at Different Stages of the Appraisal Process

**Figure 3:**
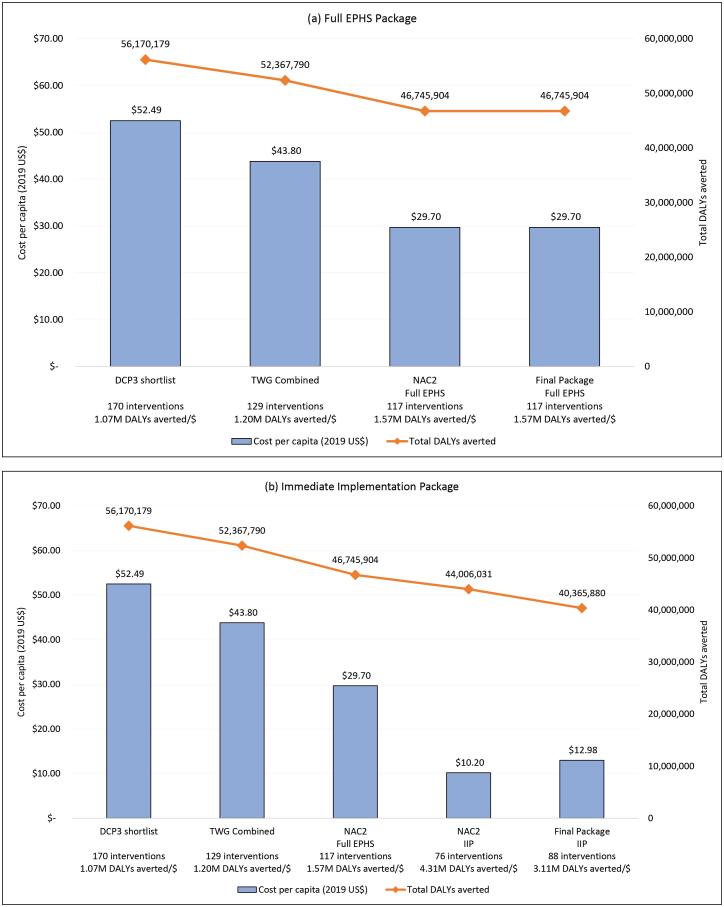
Costs Per Capita, DALYs Averted, Number of Interventions and Millions of DALYs Averted Per US Dollar Spent by Stage in the Deliberation Process for (a) Full EPHS Package and (b) Immediate Implementation Package. Abbreviations: DALYs, disability-adjusted life years; EPHS, essential package of health services; DCP3, Disease Control Priorities 3; IIP, immediate implementation package; NAC, National Advisory Council; TWG, technical working group.

Figure 3 shows the progression in total costs per capita and DALYs averted of the prioritised set of interventions throughout the different stages of the appraisal process. The figure also shows the total number of interventions and the number of DALYs averted per dollar per capita spent.

 The initial shortlist of 170 interventions had an estimated cost per capita of US$ 52.49 and could be expected to avert 56.17 million DALYs. After the TWG meetings, 129 interventions were considered to be of high priority, at a cost of US$ 43.80 per capita, averting 52.37 million DALYs. At NAC2, a full EPHS was proposed, composed of 117 interventions at US$ 29.70 per capita and averting 46.75 million DALYs, which was eventually approved by the UHC-EPHS SC and the IMPHC. A subset of interventions was selected at NAC2 to make up the IIP, composed initially of 76 interventions, at US$ 10.20 and averting 44.01 million DALYs. Finally, a revised IIP, encompassing 88 interventions at a cost of US$ 12.98 per capita and averting 40.37 million DALYs, was endorsed by the UHC-EPHS SC and the IMPHC.

 The efficiency of the package evolved throughout the process. The initial 170 shortlisted interventions were estimated to avert 1.07 million DALYs per dollar per capita spent. The first stage of the appraisal process (TWG) yielded the least efficient set of prioritised interventions, expected to avert 1.20 million DALYs per dollar per capita spent. However, it is important to highlight that the aim of the initial shortlisting and the TWG was not to prioritise within a budget constraint. The most efficient package was proposed for the IIP in NAC2, projected to avert 4.31 million DALYs per dollar per capita spent, which is higher than the efficiency of the full EPHS and the final IIP, which were expected to avert 1.57 and 3.11 million DALYs per dollar per capita spent, respectively.

###  Composition of the Package Throughout the Appraisal Process

**Figure 4:**
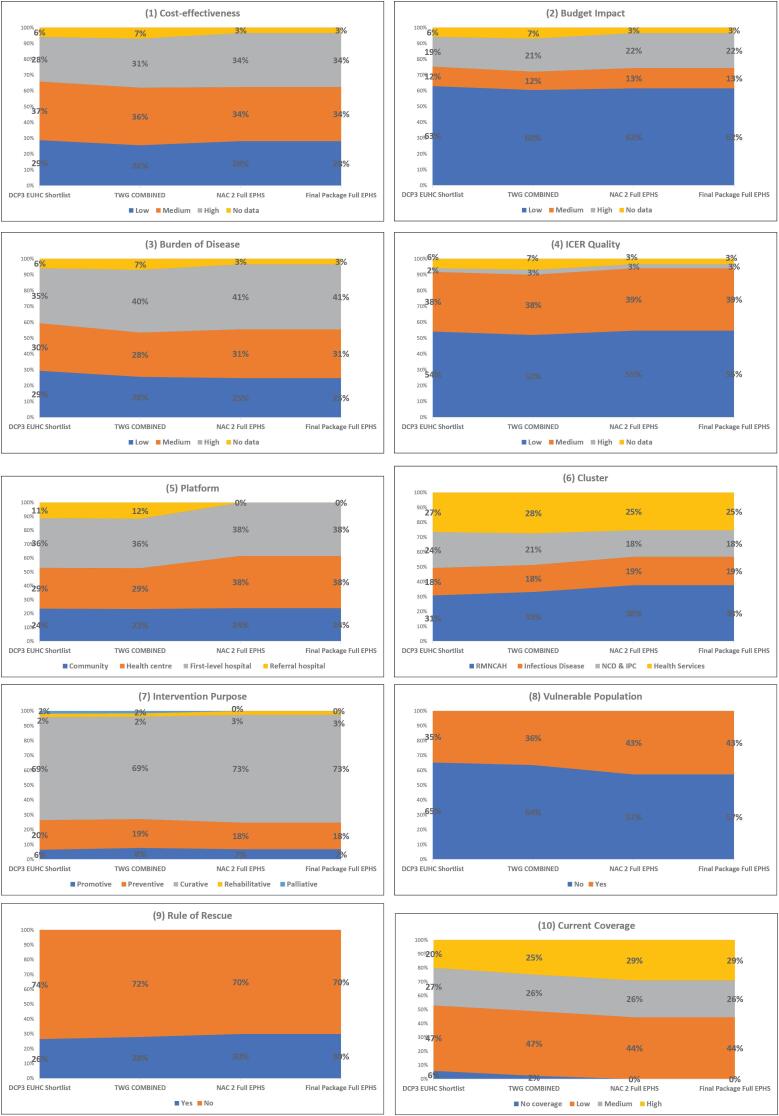
Pathway to Full EPHS Package: Distribution of Included Interventions by Stage in the Appraisal Process Broken Down by (1) Cost-Effectiveness, (2) Budget Impact, (3) Burden of Disease, (4) ICER Quality, (5) Platform, (6) Cluster, (7) Intervention Purpose, (8) Vulnerable Populations, (9) Rule of Rescue, and (10) Current Coverage. Abbreviations: EUHC, essential universal health coverage; DCP3, Disease Control Priorities 3; NAC, National Advisory Council; EPHS, essential package of health services; ICER, incremental cost-effectiveness ratio; TWG, technical working group; NCD, non-communicable disease; IPC, injury prevention and control. RMNCAH, reproductive, maternal, neonatal, child and adolescent health.

**Figure 5:**
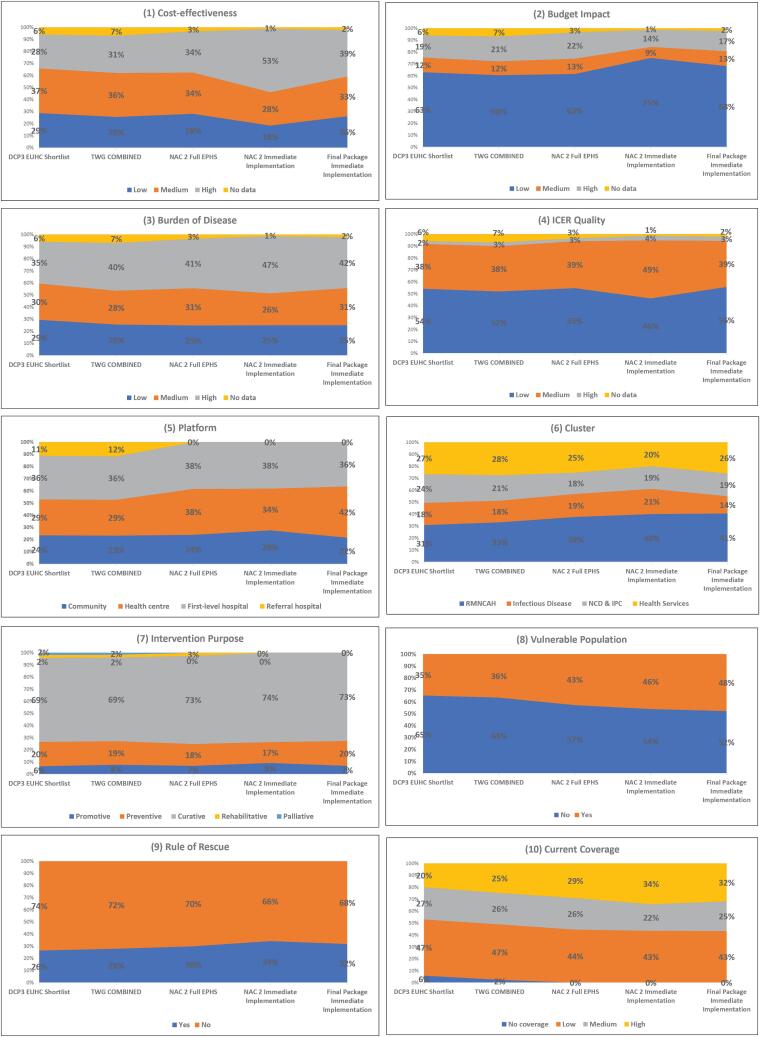
Pathway to Immediate Implementation Package: Distribution of Included Interventions by Stage in the Appraisal Process Broken Down by (1) Cost-Effectiveness, (2) Budget Impact, (3) Burden of Disease, (4) ICER Quality, (5) Platform, (6) Cluster, (7) Intervention Purpose, (8) Vulnerable Populations, (9) Rule of Rescue, and (10) Current Coverage. Abbreviations: EUHC, essential universal health coverage; DCP3, Disease Control Priorities 3; NAC, National Advisory Council; EPHS, essential package of health services; ICER, incremental cost-effectiveness ratio; TWG, technical working group; NCD, non-communicable disease; IPC, injury prevention and control.

Figures 4 and 5 show the proportion of interventions prioritised in each stage of the appraisal process broken down by decision criteria and intervention characteristics.

 The share of interventions classified as highly cost-effective increased moderately in the initial part of the process: between 28% in the DCP3 shortlisting phase to 34% in the full EPHS. However, highly cost-effective interventions made up a substantially higher share of interventions in the IIP, up to 53% of the package in the first iteration of the IIP at NAC2, then decreasing to 39% in the final iteration of the package. The share of interventions classified as having low budget impact remained constant (60%-63%) between the shortlisting phase and the full EPHS. They increased in the IIP, making up 75% of the IIP at the NAC2 and decreasing to 68% in the final iteration. Interventions preventing a high burden of disease made up the largest share of the package in all stages: from 35% in the initial shortlisting to 41% in the final EPHS. The share was highest in the first iteration of the IIP in NAC2 (47%) and dropped to 42% by the final package.

 The proportion of interventions with low ICER quality made up more than half of interventions in all stages of the appraisal process, except in the first stages of appraisal around the IIP (NAC2, where the figure dropped to 46%). Interventions with high current coverage increased from 20% in the shortlist stage to 29% in the final EPHS and further to 32% in the IIP.

 The share of interventions in the health centre platform increased throughout the process, from 29% of the initial shortlist to 38% in the final EPHS and 42% in the final IIP. Interventions based at referral hospitals made up 11% of prioritised interventions at the start of the process but were removed at NAC2 when focus changed to a district-level package of services. The proportion of RMNCAH interventions also increased steadily: from 31% in the initial shortlisting to 38% and 41% in the final EPHS package and IIP, respectively.

 The proportion of interventions involving the rule of rescue remained between 26%-34% throughout the process, reaching the highest level during the NAC2 appraisal of the IIP. Curative and preventive interventions made up the majority of prioritised interventions and their share remained steady through the process: 69%-74% and 17%-20%, respectively. Only 4% of shortlisted interventions were classified as rehabilitative or palliative; all were eliminated during the IIP appraisal stages.

###  Distribution of Interventions by Current Coverage and Cost-Effectiveness

**Figure 6:**
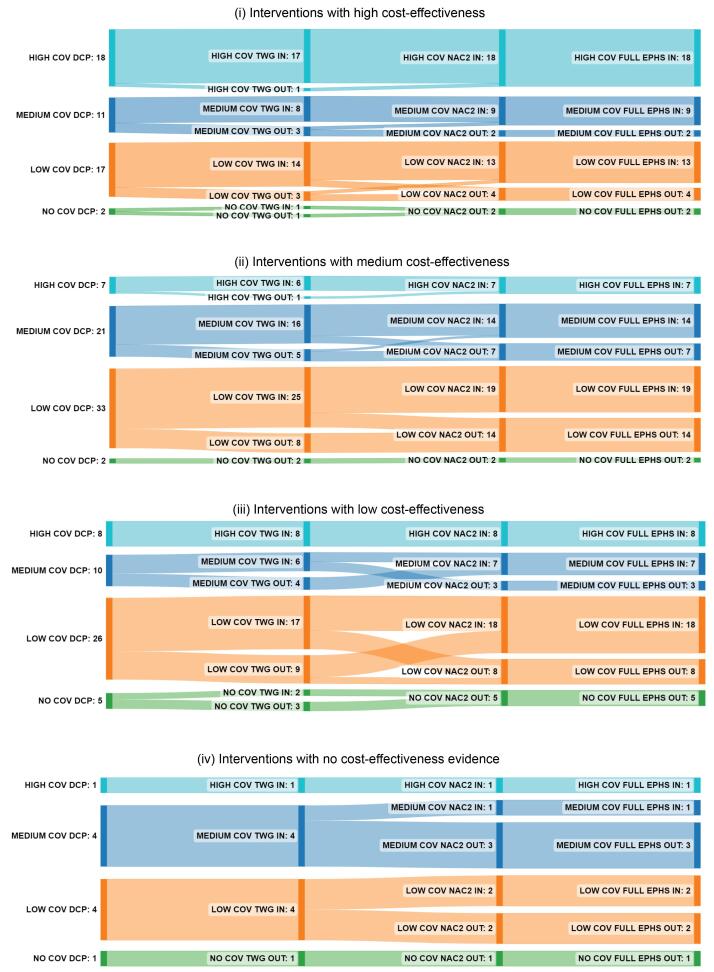
Distribution of Interventions Throughout the Process by Current Coverage and Cost-Effectiveness for the Full EPHS. Abbreviations: DCP, Disease Control Priorities; NAC, National Advisory Council; EPHS, essential package of health services; TWG, technical working group.

**Figure 7:**
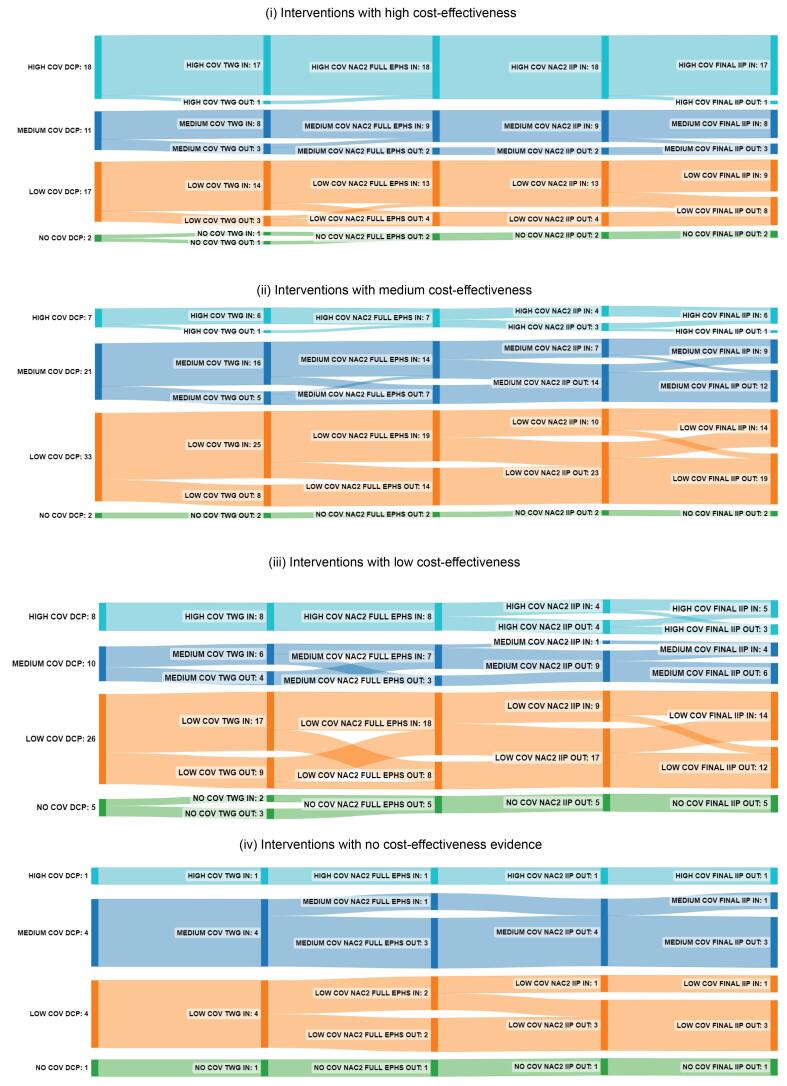
Distribution of Interventions Throughout the Process by Current Coverage and Cost-Effectiveness for the Immediate Implementation Package. Abbreviations: DCP, Disease Control Priorities; NAC, National Advisory Council; IIP, immediate Implementation Package; TWG, technical working group; EPHS, essential package of health services.

Figures 6 and 7 show the distribution of interventions broken down by cost-effectiveness and current coverage, divided between stages leading to (*a*) full EPHS and (*b*) the final IIP. In both stages, all or nearly all interventions with high coverage were included in the package, regardless of cost-effectiveness; (34/34) for the full EPHS and (28/34) for the final IIP, including 8/8 interventions with low cost-effectiveness in the full EPHS and 5/8 in the final IIP. About two-thirds (8/11) of highly cost-effective interventions with medium coverage and over half (9/17) of those with low coverage were included in the final IIP. The figure was higher in the full EPHS: 9/11 and 13/17, respectively.

## Discussion

 We have analysed the composition of the interventions included in the EPHS in Pakistan throughout different stages in the appraisal process and explored which decision criteria and intervention characteristics were valued as important at

 different stages. To our knowledge, this is the first study of its kind. A body of literature has examined decision criteria prioritisation within the context of incremental HTA in HICs. Our paper examines decision criteria and intervention characteristic prioritisation in a health-system wide reform in a lower middle-income setting, throughout the process (and, consequently, with a range of stakeholder involvement).

 We found that evidence-informed deliberative processes can lead to the design of EPHS that are progressively more affordable and efficient. The process started from a shortlist of 170 interventions at a cost per capita of US$ 52.49 and ended with an EPHS that included 117 interventions at a cost per capita of US$ 29.70, and an IIP with 88 interventions at US$ 12.98. However, cost-effectiveness was not always prioritised by stakeholders; an intermediate iteration of the package, the IIP proposed at NAC2, was more efficient than the final package. Our findings suggest that evidence uptake worked most effectively during the NAC2 IIP appraisal stages, when the share of highly cost-effective interventions prioritised was the highest in the process (53%), as was the share of interventions with low budget impact (75%) and share of interventions preventing a high burden of disease (47%).

 The TWGs were provided with intervention-specific evidence on budget impact and cost-effectiveness but not a budget constraint. They produced a list of high priority interventions which, in the aggregate, would have been unaffordable. Substantial reductions in the size and cost of the package were only achieved at the NAC2 stage once an explicit budget envelope was stipulated and stakeholders began to engage with more difficult trade-offs. However, the TWG process played a role in defining the scope of later scenarios. This initial broad prioritisation of interventions with clinical experts, who understand real-world service delivery well, was essential to arrive at packages that are not only efficient and affordable but also feasible. It is also possible that as clinical experts, TWG members have closer proximity to patients and may have been more averse to recommend discontinuation of specific interventions; clinicians’ aversion to withdrawal of existing services is well known.^19,20,24,25,42^

 Evidence from priority setting and HTA from HICs have found that cost-effectiveness is the key predictor of the decision to adopt a new intervention.^10-12,14-18,43^ In all cases, this evidence comes from incremental priority setting, rather than whole sector package revision. Other factors also appear to predict adoption to a lesser degree, including burden of disease,^13,16,18^ availability of alternative interventions,^43^ the quality, volume and recency of evidence available,^11,14,17^ the level of uncertainty of the evidence^16^ and affordability.^18^ It is challenging to directly situate our findings with these results from HICs; in our analysis we do not compare the relative importance of different criteria in one stage of the process but rather explore the importance of a criterion across appraisal stages.

 However, our findings suggest both similarities and differences. Stakeholders appeared to favour highly cost-effective interventions, but not uniformly, with highly cost-effective interventions making up between 28% and 53% of the IIP at different stages and improving when budget constraints were introduced. Stakeholders appeared to consistently favour interventions with low budget impact and those which prevented a high burden of disease (making up 60%-75% and 35%-47% of the IIP at different stages, respectively). Understanding what decision criteria and intervention characteristics are most important to different types of stakeholders throughout the process may inform researchers and others on how to target their technical support. Such insights may suggest areas where methodological developments would be most useful and where greater precision in context-specific estimates is needed.

 Barriers to disinvestment in healthcare are well documented. The evidence points towards both political barriers^22,24,42,44^ as well as barriers related to the unavailability of structured decision-making processes, the difficulty of identifying potential candidates for disinvestment, and the lack of relevant evidence with which to make informed decisions.^19-23^ However, the EPHS design process, using the DCP3 framework, provided a formal process and relevant evidence yet disinvestment from comparatively less cost-effective interventions was not always achieved. The reasons for this are not clear. Stakeholders appeared to prioritise interventions with high current coverage rather than those representing comparatively better value for money. This pattern could suggest an aversion to disinvestment at the expense of efficiency; in other words, averting fewer DALYs appears to have been preferred over withdrawal of existing services. This could potentially be due to political concerns. However, it could also reveal legitimate concerns about feasibility and a certain degree of risk aversion: interventions with high current coverage have already proven to be feasible at scale; those with low or no coverage have not. Furthermore, several other criteria were considered throughout the process, including equity and financial risk protection but, due to a lack of reliable data, these criteria were assessed qualitatively. It is possible that stakeholders traded off cost-effective interventions for those interventions they may have considered more favourable for reasons of equity and financial risk, which would not have been captured in our analyses. It remains hard to disentangle a primary reason to explain why cost-effectiveness was not always prioritised. Further research should systematically study the relationship between decision criteria, to better understand the trade-offs required from stakeholders involved in the prioritisation process.

 The aim of the EPHS design process in Pakistan was to arrive at an actionable package. This requires linking decisions to investment plans and financing systems and providing operational guidance on how existing expenditures can be allocated within the available fiscal space.^45^ As a result, the evidence-informed deliberative process was framed largely around considerations of cost and cost-effectiveness. The use of scenarios in the latter appraisal stages highlighted difficult trade-offs faced at a health system level, forcing stakeholders to confront the value for money of interventions prioritised and deprioritised. However, it remains unclear whether the relative importance of cost-effectiveness seen here is an artifact of how the process was framed or whether it reflects the values by policy-makers involved in priority setting.

 The relative importance of decision criteria and intervention characteristics in the EPHS design process and appraisal is largely understudied, particularly in LMICs. Stakeholders and members of society as a whole have different interests and, as such, may reasonably have differing views on which values should guide priority setting.^36^ It is then unsurprising that decisions made by different sets of stakeholders reflect different priorities, such as feasibility, technical efficiency and political acceptability. While formal techniques such as multi-criteria decision analysis weigh decision criteria explicitly and quantitatively, it is not clear whether more qualitative methods, such as the ones employed in Pakistan, result in an EPHS that most accurately reflect the values of the population (vis-à-vis those of stakeholders). We encourage others to carry out studies throughout the EPHS design process in other LMIC settings, and to further reflect on how the framing of the process and involvement of different stakeholders influence the decision criteria prioritised and, ultimately, the shape of the package.

###  Limitations

 This study has several limitations. We were not able to analyse the complete process as we did not have all the assessment criteria for all 218 DCP3 EUHC package interventions, given that the initial scoping reduced the assessment to 170 interventions. Further, while we know which of the 218 DCP3 EUHC interventions were ultimately excluded from the package (and are therefore able to understand the opportunity cost of the decisions made) we do not know whether there are any other currently implemented interventions (outside of DCP3) which will no longer be offered once the EPHS is rolled out. Achieving clarity on this point would allow a better understanding of the incremental difference in cost and effectiveness between the existing health offer and the EPHS.

 While we were able to examine some potential drivers of decision-making, such as cost-effectiveness, other drivers, like feasibility, could not be quantitatively included in our analysis. Relatedly, and unfortunately, we did not carry out qualitative research with stakeholders. Such work would have allowed us to further understand the political economy of the decision-making process and help us interpret how and why stakeholders prioritised certain decision criteria and intervention characteristics.

 Future analyses should consider a broader set of drivers of adoption and methodological work should be developed to facilitate their assessment. As we summarise the results along different stages of the process, it should be noted that our cost and cost-effectiveness data have limitations,^34,35^ including that values used are point estimates and do not contain information on the range of uncertainty around the parameter means. Data on current coverage was compiled by members of Health Planning, System Strengthening & Information Analysis Unit and only available during the latter stages of the appraisal; however, broad estimates of coverage were also included in a mapping exercise available to stakeholders prior to the appraisal.^46^ Lastly, our analysis does not statistically ascertain the correlation between specific decision criteria and the decision outcome, due to the overlap between the different criteria and other confounding factors.

## Conclusion

 We summarise the process of prioritising and deprioritising interventions in the EPHS design process in Pakistan. The composition of the package changed; efficiency and affordability generally increased throughout the process, although not uniformly. Stakeholders largely prioritised interventions with low budget impact and those preventing a high burden of disease and higher current coverage. Highly cost-effective interventions were not uniformly prioritised. Overall, by involving different types of stakeholders in the process, a range of criteria and values were considered such as efficiency, feasibility and acceptability.

## Acknowledgements

 The authors would like to the MNHSR&C of Pakistan and the Health Planning, Systems Strengthening and & Information Analysis Unit for their collaboration in obtaining all the data necessary to carry out the analysis.

## Ethical issues

 Ethical approvals were obtained from the London School of Hygiene and Tropical Medicine (21247) and Aga Khan University (2019-1992-5190); MoH clearance is being sought.

## Competing interests

 Authors declare that they have no competing interests.

## Funding

 This paper is part of a series of papers coordinated by the DCP3 Country Translation Project at the London School of Hygiene and Tropical Medicine, which is funded by the Bill & Melinda Gates Foundation (OPP1201812). The sponsor had no involvement in paper design; collection, analysis and interpretation of the data; and in the writing of the paper.

## Supplementary files

General Appendix contains Figure S1, Box S1, and Tables S1 and S2.

Supplementary file 1. Values Used for Each Intervention for Each Decision Criteria (Evidence and Evidence Quality).

Supplementary file 2. Values Used for Each Intervention Characteristic.

Supplementary file 3. Status of Intervention Per Stage in the Deliberation Process.
