# Supplementary material for: The Use of Evidence to Design an Essential Package of Health Services in Pakistan: A Review and Analysis of Prioritisation Decisions at Different Stages of the Appraisal Process
Source: Int J Health Policy Manag. 2024 Mar 9;13:8043. doi: 10.34172/ijhpm.2024.8043 (PMC11608344; doi:10.34172/ijhpm.2024.8043)
Supplement: Supplementary file 2 — Supplementary file 1. Values Used for Each Intervention for Each Decision Criteria (Evidence and Evidence Quality). [file ijhpm-13-8043-s002.pdf]

**Supplementary Information**

**Article title: The use of evidence to design an essential package of health services in Pakistan: a review and analysis of prioritisation decisions at different stages of the appraisal process**

**Journal name:** International Journal of Health Policy and Management (IJHPM)

**Authors' information**: Sergio Torres-Rueda[1], Anna Vassall[1]*, Raza Zaidi[2], Nichola Kitson[1], Muhammad Khalid[2], Wahaj Zulfiqar[2], Maarten Jansen[3], Wajeeha Raza[4], Maryam Huda[5], Frank Sandmann[6], Rob Baltussen[3], Sameen Siddiqi[5], Ala Alwan[7]

1. Department of Global Health & Development, London School of Hygiene and Tropical Medicine, London, UK

2. Ministry of National Health Services, Regulations and Coordination, Islamabad, Pakistan

3. Department of Health Evidence, Radboud Institute of Health Sciences, Radboud University Medical Center, Nijmegen, The Netherlands

4. Centre for Health Economics, University of York, York, UK

5. Department of Community Health Sciences, Aga Khan University, Karachi, Pakistan

6. Department of Infectious Disease Epidemiology, London School of Hygiene and Tropical Medicine, London, UK

7. DCP3 Country Translation Project, London School of Hygiene and Tropical Medicine, London, UK

* Corresponding author: anna.vassall@lshtm.ac.uk

**Supplementary File 1**

**Supplementary Table S1:** Values used for each intervention for each decision criteria (evidence and evidence quality)

**Abbreviations:** DCP, Disease Control Priorities; EUHC, Essential Universal Health Coverage; TWG, technical working group; NAC, National Advisory Council; EPHS, Essential Package of Health Services; IIP, immediate implementation package; World Health Organization; HiB, Haemophilus influenzae type b; BCG, Bacillus Calmette-Guéri; IRS, indoor residual spraying; HPV, human papillomavirus; STIs, sexually transmitted infections; TB, tuberculosis; WASH, water, sanitation and hygiene; G6PD, glucose-6-phosphate dehydrogenase; BEmONC, Basic Emergency Obstetric and Newborn Care; PLHIV, people living with HIV; IMAI, integrated management of adolescent and adult illness; CVD, cardiovascular disease; COPD, chronic obstructive pulmonary disease; ACEi, angiotensin-converting enzyme inhibitor; ARBs, angiotensin receptor blockers; IV, intravenous; CEmONC, Comprehensive Emergency Obstetric and Newborn Care; ART, antiretroviral treatment; NTDs, neglected tropical diseases; ECD, early childhood development; ARV, antiretroviral; IMCI, Integrated management of childhood illness; IUCDs, IDU, intravenous drug user; MSM, men who have sex with men; MDR, multidrug-resistant tuberculosis; XDR, extensively drug-resistant tuberculosis; C, community; HC, health centre; FLH, first-level hospital; RH, referral hosptal; ICER, incremental cost-effectiveness ratio

| DCP3 Code | Intervention name | Cost-effectiveness | Budget impact | Burden of disease | ICER quality | Current coverage |
|---|---|---|---|---|---|---|
| C1 | Antenatal and postpartum education on birth spacing | High | Low | Medium | Medium | High |
| C10 | Education on handwashing, personal hygiene and safe disposal of children’s stool | High | Low | High | Medium | High |
| C11 | Pneumococcus vaccination | Medium | Medium | High | High | High |
| C12 | Rotavirus vaccination | Medium | Low | High | High | High |
| C14 | Vitamin A and zinc for children | Medium | High | High | Low | High |
| C16 | Childhood vaccination series (diphtheria, pertussis, tetanus, polio, BCG, measles, hepatitis B, HiB, rubella) | High | Medium | High | Medium | High |
| C17 | Indoor residual spraying | Medium | Medium | High | Medium | Low |
| C18 | Education of schoolchildren on oral health | Medium | High | Medium | Low | Low |
| C19 | Vision pre-screening by teachers; vision tests and provision of ready-made glasses on-site by eye specialists | Medium | High | High | Low | Low |
| C2 | Counselling of mothers on providing thermal care for pre- term new-borns (delayed bath and skin to skin contact) | High | Low | Medium | Medium | Low |
| C20 | School based HPV vaccination for girls | Low | Low | Low | High | No |
| C21 | Mass drug administration (NTDs) | Low | High | Medium | Low | No |
| C23 | Adolescent-friendly services for STIs | Medium | Low | Medium | Low | Low |
| C24 | Life skills training in schools | Low | High | High | Low | Low |
| C27a | Provision of iron and folic acid supplementation to pregnant women, and provision of food or caloric supplementation to pregnant women in food-insecure households | High | High | High | Medium | High |

| DCP3 Code | Intervention name | Cost-effectiveness | Budget impact | Burden of disease | ICER quality | Current coverage |
|---|---|---|---|---|---|---|
| C27b | Provision of iron and folic acid supplementation to pregnant women, and provision of food or caloric supplementation to pregnant women in food-insecurity households | High | High | High | Medium | High |
| C28 | Community-based HIV testing and counselling (for example, mobile units and venue-based testing), with appropriate referral or linkage to care and | High | Low | Low | Medium | Low |
| C30a | Provision of condoms to key populations, including sex workers, men who have sex with men, people who inject drugs, transgender populations, and prisoners | High | Low | Medium | Medium | Low |
| C30b | Provision of Disposable syringes who inject drugs (IDU) | High | Low | Medium | Medium | Low |
| C32 | Routine contact tracing to identify individuals exposed to TB and link them to care | Low | Low | Low | Low | High |
| C33 | Test for G6PD deficiency | Low | Low | Low | Low | Medium |
| C3a | Management of labour and delivery in low-risk women by skilled attendant (CL) | High | Low | High | Low | Low |
| C3b | Basic neonatal resuscitation following delivery (CL) | High | Low | High | Low | Low |
| C3c | Management of labour and delivery in low-risk women by skilled attendant (PHC) | High | Low | High | Low | High |
| C3d | Basic neonatal resuscitation following delivery (PHC) | High | Low | High | Low | High |
| C34 | Environmental management for malaria | High | Medium | High | Low | Low |
| C4 | Promotion of breastfeeding and complementary feeding by community health workers | High | Low | High | Medium | High |
| C41 | Mass drug administration (malaria) | High | High | High | Low | No |
| C43 | Early detection and treatment of leishmaniasis, dengue, chikungunya, rabies, trachoma and helminthiasis. | Medium | Low | Medium | Low | Medium |
| C45 | Identify and refer patients with high risk | None | None | None | None | High |
| C46 | In the context of an emerging infectious outbreak, provide advice and guidance on how to recognize early symptoms and signs and when to seek medical attention | Medium | Low | Medium | Low | High |
| C47 | Exercise-based pulmonary rehabilitation | Medium | Low | Low | Low | No |
| C48 | Self-managed treatment of migraine | Low | Low | Medium | Low | No |
| C5 | Tetanus toxoid immunization among schoolchildren and women attending antenatal care | High | Low | Medium | Medium | High |
| C50 | Parent training of high-risk families, including nurse home visitation for child maltreatment | Medium | High | High | Low | Low |
| C51 | WASH behaviour change interventions, such as community led total sanitation | Low | Medium | Medium | Low | High |
| C53a | Identification/screening of the early childhood development issues motor, sensory and language stimulation | Medium | High | High | Low | Low |
| C53b | ECD rehabilitation interventions | Medium | High | High | Low | Low |
| C56 | Interventions for wheelchair users | None | None | None | None | No |
| C8 | Acute severe malnutrition management | Low | High | High | Low | High |
| C9 | Integrated community case management | Medium | Low | Low | Low | Low |
| FLH1 | Care for foetal growth restriction | Medium | High | High | Low | Medium |
| FLH10 | Surgical termination of pregnancy by maternal vacuum aspiration and dilatation & curettage | Low | Low | Low | Low | Low |
| FLH11 | Care for severe childhood infections | Medium | High | High | Low | Medium |
| FLH12 | Severe acute malnutrition management | Medium | Low | Low | Medium | Medium |
| FLH13 | Early detection and treatment of early-stage cervical cancer | Medium | Low | Low | Medium | Low |
| FLH14 | Insertion and removal of contraceptives | Medium | Low | Medium | Low | Low |
| FLH15 | Tubal ligation | Low | High | High | Low | Low |
| FLH16 | Vasectomy | Medium | Low | Medium | Low | Low |

| DCP3 Code | Intervention name | Cost-effectiveness | Budget impact | Burden of disease | ICER quality | Current coverage |
|---|---|---|---|---|---|---|
| FLH17 | Referral of cases of treatment failure for drug susceptibility testing; enrolment of those with MDR-TB for treatment per WHO guidelines (either short- or long-term regimen) | High | Low | High | Low | High |
| FLH18 | Evaluation and management of fever in clinically unstable individuals using WHO IMAI guidelines, including empiric parenteral antimicrobials and antimalarial and resuscitative measures for septic shock | High | Low | High | Medium | Medium |
| FLH2 | Induction of labour post-term | Medium | Low | Medium | Low | Medium |
| FLH20 | Management of acute coronary syndromes | Low | High | Medium | Low | Medium |
| FLH22 | Management of acute exacerbations of asthma and COPD using systemic steroids, inhaled beta-agonists and if indicated oral antibiotics and oxygen therapy | Low | High | Medium | Medium | Low |
| FLH23 | Medical management of acute heart failure | Medium | Low | High | Medium | Medium |
| FLH24 | Bowel obstruction management | High | Low | Medium | Low | Medium |
| FLH25 | Calcium and vitamin D supplementation for secondary prevention of osteoporosisE264 | Low | High | High | Low | Low |
| FLH26 | Combination therapy, including low-dose corticosteroids and generic disease-modifying antirheumatic drugs (including methotrexate), for individuals with moderate to severe rheumatoid arthritis | High | Low | Medium | Low | Medium |
| FLH27 | In settings where sickle cell disease is a public health concern, universal newborn screening followed by standard prophylaxis against bacterial infections and malaria | Medium | Low | Low | Medium | Low |
| FLH28 | In setting where specific single-gene disorders are a public health concern (for example thalassemia), retrospective identification of carriers plus prospective (premarital) screening and counselling to reduce rates of conception | Medium | Low | Low | Low | Low |
| FLH3 | Jaundice management with phototherapy | Medium | Low | Low | Low | Medium |
| FLH30 | Intoxication/poisoning management | Medium | Low | Low | Low | Low |
| FLH31 | Appendectomy | Low | Medium | Medium | Medium | High |
| FLH32 | Assisted vaginal delivery using vacuum extraction or forceps | Medium | Low | Medium | Medium | Low |
| FLH33 | Craniotomy for trauma | Medium | Low | Low | Medium | Low |
| FLH34 | Colostomy for acute bowel obstruction/volvulus and injuries. | High | Low | Medium | Low | Medium |
| FLH35 | Escharotomy or fasciotomy | Medium | Low | High | Medium | Low |
| FLH36 | Management of non-displaced fractures | High | Low | High | Medium | Medium |
| FLH37 | Hernia Repair | Medium | Low | Medium | Medium | Medium |
| FLH38 | Hysterectomy for uterine rupture or intractable postpartum haemorrhage | High | Low | Medium | Medium | High |
| FLH39 | Irrigation and debridement of open fractures | Medium | Low | Medium | Low | Medium |
| FLH4 | Eclampsia management with magnesium sulphate, including initial stabilization at health centres | Medium | Low | High | Medium | High |
| FLH40 | Management of osteomyelitis, including surgical debridement | Medium | Medium | High | Low | Medium |
| FLH41a | Management of Septic Arthritis | Medium | Low | Low | Medium | Low |
| FLH41b | Placement of External Fixation and Use of Traction for Fractures | Medium | Low | Low | Medium | Low |
| FLH42 | Relief of urinary obstruction by catheterization for fractures | High | Low | High | Low | High |
| FLH43 | Removal of gallbladder, including emergency surgery | Medium | Low | Medium | Low | Medium |
| FLH44 | Repair of perforations (for example perforated peptic ulcer, typhoid ileal perforation) | High | Low | High | Low | High |
| FLH45 | Resuscitation with advanced measures | Medium | Low | Medium | Low | Low |
| FLH46 | Basic Skin grafting | Medium | Low | Low | Medium | Low |
| FLH48a | Trauma laparotomy | High | Low | High | Medium | High |

| DCP3 Code | Intervention name | Cost-effectiveness | Budget impact | Burden of disease | ICER quality | Current coverage |
|---|---|---|---|---|---|---|
| FLH49 | Trauma-related amputations | High | Low | High | Low | Medium |
| FLH5 | Maternal sepsis management | Medium | Low | Medium | Medium | Medium |
| FLH50 | Tube thoracostomy | Medium | Low | Low | Low | Medium |
| FLH52 | Compression therapy for amputations, burns, and vascular or lymphatic disorders | Medium | Low | Low | Medium | Medium |
| FLH53 | Evaluation and acute management of swallowing dysfunction | Medium | Low | Low | Low | Medium |
| FLH57 | Prevention and relief of refractory suffering and acute pain related to surgery, serious injury or other serious, complex or life-limiting health problems | None | None | None | None | Low |
| FLH58 | First level hospital pathology services | None | None | None | None | Medium |
| FLH6 | Management of new-born complications infections, meningitis, septicaemia, pneumonia and other very serious infections requiring continuous supportive care (such as IV fluids and oxygen) | High | Low | High | Medium | Medium |
| FLH7 | Preterm labour management | Low | Medium | Low | Medium | Medium |
| FLH8 | Management of labour and delivery in high-risk women, including operative delivery (CEmONC) | Low | High | High | Low | Medium |
| FLH9 | Surgery for ectopic pregnancy | High | Low | Medium | Medium | Medium |
| HC1 | Early detection and treatment of neonatal pneumonia with oral antibiotics | High | Low | High | Medium | High |
| HC10 | Screening and management of diabetes (gestational diabetes or pre-existing type II diabetes) | Low | Low | Low | Medium | Low |
| HC11 | Management of labour and delivery in low-risk women (BEmONC), including initial treatment of obstetric or delivery complications prior to transfer (Also included in Surgery package of services) | High | High | High | Low | High |
| HC12 | Detection and treatment of childhood infections with danger signs (IMCI) | High | High | High | Low | Low |
| HC13 | Among all individuals who are known to be HIV+, immediate ART initiation with regular monitoring of viral load for | Low | Low | Low | Medium | Low |
| HC14 | Psychological treatment | Low | High | Medium | Medium | Low |
| HC16 | Post gender-based violence care | Low | Medium | Medium | Low | Low |
| HC17 | Syndromic management of common sexual and reproductive tract infections (for example urethral discharge, genital ulcer and others) | Medium | High | High | Low | High |
| HC19 | For individuals testing positive for hepatitis B and C, assessment of treatment eligibility by trained providers followed by initiation and monitoring of ART when indicated | Low | High | High | Medium | Low |
| HC2 | Miscarriage and abortions management | Low | Low | Medium | Low | Medium |
| HC20 | Hepatitis B and C testing of high-risk individuals identified in the national testing policy with appropriate referral of positive individuals to trained providers | Medium | Medium | Medium | Low | Low |
| HC21 | Partner notification and expedited treatment for common STIs including HIV | High | Medium | High | Medium | Medium |
| HC23 | Provider-initiated testing and counselling for HIV, STIs and hepatitis for all in contact with the health system in high- prevalence setting, including prenatal care with appropriate referral/ linkages to care including immediate ART initiation for those testing positive for HIV | Medium | High | High | Medium | Medium |
| HC24 | Hepatitis B vaccination for high-risk populations, including healthcare workers, IDU, MSM, household contacts and partners with multiple sex partners | Medium | Low | Low | Medium | Low |
| HC25 | Medical male circumcision | Medium | Medium | Medium | Low | Low |

| DCP3 Code | Intervention name | Cost-effectiveness | Budget impact | Burden of disease | ICER quality | Current coverage |
|---|---|---|---|---|---|---|
| HC26 | For PLHIV and children under five who are close contacts or household members of individuals with active TB, perform symptom screening and chest radiograph; if there is no active TB, provide isoniazid preventive therapy according to current WHO guidelines | High | Medium | High | Medium | Low |
| HC27 | Diagnosis of TB and first-line treatment | High | Low | High | Low | High |
| HC28 | Screening for HIV in all individuals with a diagnosis of active TB; if HIV infection is present, start (or refer for) ARV treatment and HIV care | High | Low | Medium | Low | Low |
| HC29 | Latent-TB screening and IPT for PLHIV | High | Low | Medium | Low | Low |
| HC3 | Management of premature rupture of membranes, including administration of antibiotics | Low | Low | Low | Low | Low |
| HC30 | Fever management for clinically stable | Medium | Low | Low | Low | High |
| HC32 | Provision of insecticide nets to U5 children and pregnant women attending health centres | High | Low | Medium | Medium | Medium |
| HC33 | Identify and refer for progressive illness ** | None | None | None | None | Medium |
| HC36 | Long-term combination therapy for persons with multiple CVD risk factors, including screening for CVD in community setting using non-lab-based tools to assess overall CVD risk | Low | Medium | Medium | Low | Low |
| HC37 | Low-dose inhaled corticosteroids and bronchodilators for asthma and for selected patients with COPD | Low | Low | Low | Medium | Low |
| HC38 | Provision of aspirin for all cases of suspected acute myocardial infarction | Medium | Low | Low | High | Low |
| HC39a | Screening and ACEi or ARBs for kidney disease | Low | Low | Low | Low | Low |
| HC41 | Secondary prophylaxis for rheumatic fever | Low | Low | Low | Low | Low |
| HC42 | Treatment of acute pharyngitis for rheumatic fever | High | Low | Medium | Low | High |
| HC45 | Opportunistic screening for hypertension | Medium | High | High | Medium | Low |
| HC46 | Tobacco cessation counselling | High | High | High | Medium | No |
| HC48 | Support for caregivers of dementia patients | Medium | Low | Low | Medium | No |
| HC49 | Bipolar disorder management | Low | Medium | Low | Low | No |
| HC4a | Provision of condoms and hormonal contraceptives, including emergency contraceptives | High | Low | High | Medium | Low |
| HC4b | Provision of condoms and hormonal contraceptives, including insertion and removal of contraceptives (PHC) | High | Low | High | Medium | Low |
| HC50 | Management of depression and anxiety disorders with psychological and generic antidepressants therapy | Low | High | Medium | Low | Low |
| HC53 | Screening and brief alcohol intervention | Low | Low | Low | Low | No |
| HC55 | Primary prevention of osteoporosis | Low | Low | Medium | Low | Medium |
| HC56 | Screening for congenital hearing loss | Low | Medium | Medium | Medium | Low |
| HC57a | Dental extraction (PHC) | Medium | High | High | Low | Medium |
| HC57b | Dental extraction (FLH) | Medium | High | High | Low | Medium |
| HC58a | Drainage of dental abscess (PHC) | Low | High | Medium | Low | Low |
| HC59 | Drainage of superficial abscess | Low | Medium | Medium | Low | Medium |
| HC5a | Counselling on kangaroo care for new-borns (CL) | Medium | Low | Low | Low | Low |
| HC5b | Counselling on kangaroo care for new-borns (PHC) | Medium | Low | Low | Low | Low |
| HC6 | Management of neonatal sepsis, pneumonia and meningitis using injectable and oral antibiotics | High | Low | Low | Medium | Low |
| HC60 | Non-displaced fractures management | Low | Low | Low | Low | Low |
| HC61 | Resuscitation with basic life support measures | None | None | None | None | Low |

| DCP3 Code | Intervention name | Cost-effectiveness | Budget impact | Burden of disease | ICER quality | Current coverage |
|---|---|---|---|---|---|---|
| HC62 | Suturing laceration | Low | Low | Low | Low | Medium |
| HC63a | Treatment of caries (PHC) | Low | High | Medium | Low | Low |
| HC64 | Basic management of MNIs and disorders | Low | Low | Low | Low | Low |
| HC66 | Psychosocial support and counselling | Low | Low | Low | Low | Low |
| HC67 | Expanded palliative care and pain control measures, including prevention and relief of all physical and psychological symptoms of suffering | None | None | None | None | Low |
| HC68 | Health centre pathology services ** | None | None | None | None | Low |
| HC7 | Pharmacological termination of pregnancy | High | Low | High | Low | Low |
| HC9a | Screening of hypertensive disorders in pregnancy | Low | Low | Low | Low | High |
| HC9b | Screening and management of hypertensive disorders in pregnancy | Low | Low | Low | Low | High |
| P5 | Systematic identification of individuals with TB symptoms among high-risk groups and linkages to care (active case finding) | Low | Medium | Medium | Medium | High |
| RH1 | Full supportive care for preterm new-borns | High | Low | High | Medium | Medium |
| RH2 | Specialized TB services, including management of MDR- and XDR-TB treatment failure and surgery for TB | Medium | Low | Medium | Low | Medium |
| RH3 | Management of refractory febrile illness including etiologic diagnosis at reference microbial laboratory | Medium | Low | Low | Low | Medium |
| RH4 | Management of acute ventilator failure due to acute exacerbations of asthma and COPD | Low | Low | Low | Medium | Low |
| RH5 | Retinopathy screening via telemedicine, followed by treatment using laser photocoagulation | Medium | Low | Low | Medium | Low |
| RH6 | Use of percutaneous coronary intervention for acute myocardial infarction where resources permit | Low | Medium | High | Low | Low |
| RH7 | Treatment of early-stage breast cancer with appropriate multimodal approaches (including generic chemotherapy) with curative intent for cases detected by clinical examination | Low | Low | Low | Medium | Medium |
| RH8 | Treatment of early-stage colorectal cancer with appropriate multimodal approaches (including generic chemotherapy) with curative intent for cases detected by clinical examination | Low | Low | Low | Medium | Low |
| RH9 | Treatment of early-stage childhood cancers (such as Burkitt and Hodgkin lymphoma, acute lymphoblastic leukaemia, retinoblastoma and Wilms tumour) with curative intent in paediatric cancer units or hospitals | Low | Low | Medium | Medium | Low |
| RH10 | Elective surgical repair of common orthopaedic injuries (for example meniscal and ligamentous tears) in individuals with severe functional limitation | High | Medium | High | Medium | Low |
| RH11 | Urgent, definitive surgical management of orthopaedic injuries (for example open reduction and internal fixation) | Medium | Low | High | Medium | Low |
| RH12 | Repair of cleft lip and cleft palate | Low | Low | Low | Low | Medium |
| RH13 | Repair of club foot | High | Low | Medium | Low | Low |
| RH14 | Cataract extraction | Low | Medium | High | Medium | High |
| RH15 | Repair of anorectal malformations and Hirschsprung's disease | Medium | Low | Low | Low | Low |
| RH16 | Repair of obstetric fistula | Medium | Low | Medium | Low | Low |
| RH17 | Ventriculoperitoneal Shunt | Medium | Low | Low | Low | Low |
| RH18 | Surgery for Trachomatous Trichiasis | Medium | Low | Medium | Medium | Medium |

| DCP3 Code | Intervention name | Cost-effectiveness | Budget impact | Burden of disease | ICER quality | Current coverage |
|---|---|---|---|---|---|---|
| RH19 | Referral level hospital pathology services | None | None | None | None | Medium |
| RH20 | Speciality pathology services | None | None | None | None | Medium |
