# Supplementary material for: The Use of Evidence to Design an Essential Package of Health Services in Pakistan: A Review and Analysis of Prioritisation Decisions at Different Stages of the Appraisal Process
Source: Int J Health Policy Manag. 2024 Mar 9;13:8043. doi: 10.34172/ijhpm.2024.8043 (PMC11608344; doi:10.34172/ijhpm.2024.8043)
Supplement: Supplementary file 3 — Supplementary file 2. Values Used for Each Intervention Characteristic. [file ijhpm-13-8043-s003.pdf]

Supplementary Information

Article title: The use of evidence to design an essential package of health services in Pakistan: a review and analysis of prioritisation decisions at different stages of the appraisal process

Journal name: International Journal of Health Policy and Management (IJHPM)

Authors' information: Sergio Torres-Rueda[1], Anna Vassall[1]*, Raza Zaidi[2], Nichola Kitson[1], Muhammad Khalid[2], Wahaj Zulfiqar[2], Maarten Jansen[3], Wajeeha Raza[4], Maryam Huda[5], Frank Sandmann[6], Rob Baltussen[3], Sameen Siddiqi[5], Ala Alwan[7]

1. Department of Global Health & Development, London School of Hygiene and Tropical Medicine, London, UK

2. Ministry of National Health Services, Regulations and Coordination, Islamabad, Pakistan

3. Department of Health Evidence, Radboud Institute of Health Sciences, Radboud University Medical Center, Nijmegen, The Netherlands

4. Centre for Health Economics, University of York, York, UK

5. Department of Community Health Sciences, Aga Khan University, Karachi, Pakistan

6. Department of Infectious Disease Epidemiology, London School of Hygiene and Tropical Medicine, London, UK

7. DCP3 Country Translation Project, London School of Hygiene and Tropical Medicine, London, UK

* Corresponding author: anna.vassall@lshtm.ac.uk

Supplementary File 2

Supplementary Table S2: Values used for each intervention characteristic

Abbreviations: DCP, Disease Control Priorities; EUHC, Essential Universal Health Coverage; TWG, technical working group; NAC, National Advisory Council; EPHS, Essential Package of Health Services; IIP, immediate implementation package; World Health Organization; HiB, Haemophilus influenzae type b; BCG, Bacillus Calmette-Guéri; IRS, indoor residual spraying; HPV, human papillomavirus; STIs, sexually transmitted infections; TB, tuberculosis; WASH, water, sanitation and hygiene; G6PD, glucose-6-phosphate dehydrogenase; BEmONC, Basic Emergency Obstetric and Newborn Care; PLHIV, people living with HIV; IMAI, integrated management of adolescent and adult illness; CVD, cardiovascular disease; COPD, chronic obstructive pulmonary disease; ACEi, angiotensin-converting enzyme inhibitor; ARBs, angiotensin receptor blockers; IV, intravenous; CEmONC, Comprehensive Emergency Obstetric and Newborn Care; ART, antiretroviral treatment; NTDs, neglected tropical diseases; ECD, early childhood development; ARV, antiretroviral; IMCI, Integrated management of childhood illness; IUCDs, IDU, intravenous drug user; MSM, men who have sex with men; MDR, multidrug-resistant tuberculosis; XDR, extensively drug-resistant tuberculosis; CL, community-level; HC, health centre level; FLH, first-level hospital; RH, referral hosptal; RMNCH, Reproductive, maternal, neonatal and child health, NCD & IPC; non-communicable diseases and injury prevention and care.

DCP3 Code	Intervention name	Platform	Cluster	Vulnerable Population	Rule of rescue	Intervention Purpose
C1	Antenatal and postpartum education on birth spacing	CL	RMNCH	Yes	No	Preventive
C10	Education on handwashing, personal hygiene and safe disposal of children's stool	CL	RMNCH	Yes	No	Preventive
C11	Pneumococcus vaccination	CL	RMNCH	Yes	No	Preventive
C12	Rotavirus vaccination	CL	RMNCH	Yes	No	Preventive
C14	Vitamin A and zinc for children	CL	RMNCH	Yes	No	Preventive
C16	Childhood vaccination series (diphtheria, pertussis, tetanus, polio, BCG, measles, hepatitis B, HiB, rubella)	CL	RMNCH, Infectious Disease, NCD & IPC	Yes	No	Preventive
C17	Indoor residual spraying	CL	RMNCH, Infectious Disease	Yes	No	Preventive
C18	Education of schoolchildren on oral health	CL	RMNCH	Yes	No	Preventive
C19	Vision pre-screening by teachers; vision tests and provision of ready-made glasses on-site by eye specialists	CL	RMNCH	Yes	No	Preventive
C2	Counselling of mothers on providing thermal care for pre- term new-borns (delayed bath and skin to skin contact)	CL	RMNCH	Yes	No	Preventive

DCP3 Code	Intervention name	Platform	Cluster	Vulnerable Population	Rule of rescue	Intervention Purpose
C20	School based HPV vaccination for girls	CL	RMNCH, Infectious Disease	Yes	No	Preventive
C21	Mass drug administration (NTDs)	CL	RMNCH, Infectious Disease	Yes	No	Preventive
C23	Adolescent-friendly services for STIs	CL	RMNCH, Infectious Disease	Yes	No	Preventive
C24	Life skills training in schools	CL	RMNCH, NCD & IPC	Yes	No	Preventive
C27a	Provision of iron and folic acid supplementation to pregnant women, and provision of food or caloric supplementation to pregnant women in food-insecure households	CL	RMNCH, NCD & IPC	Yes	No	Promotive
C27b	Provision of iron and folic acid supplementation to pregnant women, and provision of food or caloric supplementation to pregnant women in food-insecurity households	HC	RMNCH, NCD & IPC	Yes	No	Promotive
C28	Community-based HIV testing and counselling (for example, mobile units and venue-based testing), with appropriate referral or linkage to care and	CL	Infectious Disease	No	No	Curative
C30a	Provision of condoms to key populations, including sex workers, men who have sex with men, people who inject drugs, transgender populations, and prisoners	CL	Infectious Disease	No	No	Promotive
C30b	Provision of Disposable syringes who inject drugs (IDU)	CL	Infectious Disease	No	No	Promotive
C32	Routine contact tracing to identify individuals exposed to TB and link them to care	CL	Infectious Disease	No	No	Curative
C33	Test for G6PD deficiency	HC	Infectious Disease	No	No	Curative
C3a	Management of labour and delivery in low-risk women by skilled attendant (CL)	CL	RMNCH	Yes	Yes	Curative
C3b	Basic neonatal resuscitation following delivery (CL)	CL	RMNCH	Yes	Yes	Curative
C3c	Management of labour and delivery in low-risk women by skilled attendant (PHC)	HC	RMNCH	Yes	Yes	Curative
C3d	Basic neonatal resuscitation following delivery (PHC)	HC	RMNCH	Yes	Yes	Curative
C34	Environmental management for malaria	CL	Infectious Disease	No	No	Preventive
C4	Promotion of breastfeeding and complementary feeding by community health workers	CL	RMNCH	Yes	No	Promotive
C41	Mass drug administration (malaria)	CL	Infectious Disease	No	No	Preventive
C43	Early detection and treatment of leishmaniasis, dengue, chikungunya, rabies, trachoma and helminthiasis.	CL	Infectious Disease	No	No	Curative
C45	Identify and refer patients with high risk	CL	Infectious Disease	No	No	Curative
C46	In the context of an emerging infectious outbreak, provide advice and guidance on how to recognize early symptoms and signs and when to seek medical attention	CL	Infectious Disease	No	No	Promotive
C47	Exercise-based pulmonary rehabilitation	CL	NCD & IPC	No	No	Rehabilitative
C48	Self-managed treatment of migraine	CL	NCD & IPC	No	No	Curative

DCP3 Code	Intervention name	Platform	Cluster	Vulnerable Population	Rule of rescue	Intervention Purpose
C5	Tetanus toxoid immunization among schoolchildren and women attending antenatal care	HC	RMNCH	Yes	No	Preventive
C50	Parent training of high-risk families, including nurse home visitation for child maltreatment	FLH	NCD & IPC	No	No	Promotive
C51	WASH behaviour change interventions, such as community led total sanitation	CL	NCD & IPC	No	No	Preventive
C53a	Identification/screening of the early childhood development issues motor, sensory and language stimulation	CL	Health Services	No	No	Rehabilitative
C53b	ECD rehabilitation interventions	HC	Health Services	No	No	Rehabilitative
C56	Interventions for wheelchair users	CL	Health Services	No	No	Preventive
C8	Acute severe malnutrition management	CL	RMNCH	Yes	No	Preventive
C9	Integrated community case management	CL	RMNCH	Yes	No	Preventive
FLH1	Care for foetal growth restriction	FLH	RMNCH	Yes	No	Curative
FLH10	Surgical termination of pregnancy by maternal vacuum aspiration and dilatation & curettage	FLH	RMNCH, Health Services	Yes	No	Curative
FLH11	Care for severe childhood infections	FLH	RMNCH	Yes	Yes	Curative
FLH12	Severe acute malnutrition management	FLH	RMNCH	Yes	Yes	Curative
FLH13	Early detection and treatment of early-stage cervical cancer	FLH	RMNCH, Infectious Disease, NCD & IPC	Yes	No	Curative
FLH14	Insertion and removal of contraceptives	FLH	RMNCH, Health Services	Yes	No	Preventive
FLH15	Tubal ligation	FLH	RMNCH, Health Services	Yes	No	Preventive
FLH16	Vasectomy	FLH	RMNCH, Health Services	Yes	No	Preventive
FLH17	Referral of cases of treatment failure for drug susceptibility testing; enrolment of those with MDR-TB for treatment per WHO guidelines (either short- or long-term regimen)	FLH	Infectious Disease	No	No	Curative
FLH18	Evaluation and management of fever in clinically unstable individuals using WHO IMAI guidelines, including empiric parenteral antimicrobials and antimalarial and resuscitative measures for septic shock	FLH	Infectious Disease	No	No	Curative
FLH2	Induction of labour post-term	FLH	RMNCH	Yes	Yes	Curative
FLH20	Management of acute coronary syndromes	FLH	NCD & IPC	No	No	Curative
FLH22	Management of acute exacerbations of asthma and COPD using systemic steroids, inhaled beta-agonists and if indicated oral antibiotics and oxygen therapy	FLH	NCD & IPC	No	Yes	Curative
FLH23	Medical management of acute heart failure	FLH	NCD & IPC	No	Yes	Curative
FLH24	Bowel obstruction management	FLH	NCD & IPC, Health Services	No	Yes	Curative

DCP3 Code	Intervention name	Platform	Cluster	Vulnerable Population	Rule of rescue	Intervention Purpose
FLH25	Calcium and vitamin D supplementation for secondary prevention of osteoporosisE264	FLH	NCD & IPC	No	No	Preventive
FLH26	Combination therapy, including low-dose corticosteroids and generic disease-modifying antirheumatic drugs (including methotrexate), for individuals with moderate to severe rheumatoid arthritis	FLH	NCD & IPC	No	No	Curative
FLH27	In settings where sickle cell disease is a public health concern, universal newborn screening followed by standard prophylaxis against bacterial infections and malaria	FLH	NCD & IPC	No	No	Preventive
FLH28	In setting where specific single-gene disorders are a public health concern (for example thalassemia), retrospective identification of carriers plus prospective (premarital) screening and counselling to reduce rates of conception	FLH	NCD & IPC	No	No	Curative
FLH3	Jaundice management with phototherapy	FLH	RMNCH	Yes	No	Curative
FLH30	Intoxication/poisoning management	FLH	NCD & IPC	No	Yes	Curative
FLH31	Appendectomy	FLH	Health Services	No	Yes	Curative
FLH32	Assisted vaginal delivery using vacuum extraction or forceps	FLH	Health Services	No	Yes	Curative
FLH33	Craniotomy for trauma	RH	Health Services	No	Yes	Curative
FLH34	Colostomy for acute bowel obstruction/volvulus and injuries.	FLH	Health Services	No	Yes	Curative
FLH35	Escharotomy or fasciotomy	FLH	Health Services	No	Yes	Curative
FLH36	Management of non-displaced fractures	FLH	Health Services	No	Yes	Curative
FLH37	Hernia Repair	FLH	Health Services	No	Yes	Curative
FLH38	Hysterectomy for uterine rupture or intractable postpartum haemorrhage	FLH	Health Services	No	Yes	Curative
FLH39	Irrigation and debridement of open fractures	FLH	Health Services	No	Yes	Curative
FLH4	Eclampsia management with magnesium sulphate, including initial stabilization at health centres	FLH	RMNCH	Yes	Yes	Curative
FLH40	Management of osteomyelitis, including surgical debridement	FLH	Health Services	No	Yes	Curative
FLH41a	Management of Septic Arthritis	FLH	Health Services	No	Yes	Curative
FLH41b	Placement of External Fixation and Use of Traction for Fractures	FLH	Health Services	No	Yes	Curative
FLH42	Relief of urinary obstruction by catheterization for fractures	FLH	Health Services	No	Yes	Curative
FLH43	Removal of gallbladder, including emergency surgery	FLH	Health Services	No	Yes	Curative
FLH44	Repair of perforations (for example perforated peptic ulcer, typhoid ileal perforation)	FLH	Health Services	No	Yes	Curative

DCP3 Code	Intervention name	Platform	Cluster	Vulnerable Population	Rule of rescue	Intervention Purpose
FLH45	Resuscitation with advanced measures	FLH	Health Services	No	Yes	Curative
FLH46	Basic Skin grafting	FLH	Health Services	No	No	Curative
FLH48a	Trauma laparotomy	FLH	Health Services	No	Yes	Curative
FLH49	Trauma-related amputations	FLH	Health Services	No	Yes	Curative
FLH5	Maternal sepsis management	FLH	RMNCH	Yes	Yes	Curative
FLH50	Tube thoracostomy	FLH	Health Services	No	Yes	Curative
FLH52	Compression therapy for amputations, burns, and vascular or lymphatic disorders	FLH	Health Services	No	No	Curative
FLH53	Evaluation and acute management of swallowing dysfunction	FLH	Health Services	No	No	Curative
FLH57	Prevention and relief of refractory suffering and acute pain related to surgery, serious injury or other serious, complex or life-limiting health problems	FLH	Health Services	No	No	Palliative
FLH58	First level hospital pathology services	FLH	Health Services	No	No	Curative
FLH6	Management of new-born complications infections, meningitis, septicaemia, pneumonia and other very serious infections requiring continuous supportive care (such as IV fluids and oxygen)	FLH	RMNCH	Yes	Yes	Curative
FLH7	Preterm labour management	FLH	RMNCH	Yes	No	Curative
FLH8	Management of labour and delivery in high-risk women, including operative delivery (CEmONC)	FLH	RMNCH, Health Services	Yes	Yes	Curative
FLH9	Surgery for ectopic pregnancy	FLH	RMNCH, Health Services	Yes	Yes	Curative
HC1	Early detection and treatment of neonatal pneumonia with oral antibiotics	HC	RMNCH	Yes	No	Curative
HC10	Screening and management of diabetes (gestational diabetes or pre-existing type II diabetes)	FLH	RMNCH, NCD & IPC	Yes	No	Curative
HC11	Management of labour and delivery in low-risk women (BEmONC), including initial treatment of obstetric or delivery complications prior to transfer (Also included in Surgery package of services)	HC	RMNCH, Health Services	Yes	Yes	Curative
HC12	Detection and treatment of childhood infections with danger signs (IMCI)	HC	RMNCH	Yes	Yes	Curative
HC13	Among all individuals who are known to be HIV+, immediate ART initiation with regular monitoring of viral load for	FLH	RMNCH, Infectious Disease	Yes	No	Curative
HC14	Psychological treatment	HC	RMNCH, NCD & IPC	Yes	No	Curative
HC16	Post gender-based violence care	HC	RMNCH, Infectious Disease	Yes	No	Rehabilitative

DCP3 Code	Intervention name	Platform	Cluster	Vulnerable Population	Rule of rescue	Intervention Purpose
HC17	Syndromic management of common sexual and reproductive tract infections (for example urethral discharge, genital ulcer and others)	HC	RMNCH, Infectious Disease	Yes	No	Curative
HC19	For individuals testing positive for hepatitis B and C, assessment of treatment eligibility by trained providers followed by initiation and monitoring of ART when indicated	FLH	Infectious Disease	No	No	Curative
HC2	Miscarriage and abortions management	HC	RMNCH	Yes	Yes	Curative
HC20	Hepatitis B and C testing of high-risk individuals identified in the national testing policy with appropriate referral of positive individuals to trained providers	HC	Infectious Disease	No	No	Curative
HC21	Partner notification and expedited treatment for common STIs including HIV	HC	Infectious Disease	No	No	Curative
HC23	Provider-initiated testing and counselling for HIV, STIs and hepatitis for all in contact with the health system in high- prevalence setting, including prenatal care with appropriate referral/ linkages to care including immediate ART initiation for those testing positive for HIV	HC	Infectious Disease	No	No	Curative
HC24	Hepatitis B vaccination for high-risk populations, including healthcare workers, IDU, MSM, household contacts and partners with multiple sex partners	FLH	Infectious Disease, NCD & IPC	No	No	Preventive
HC25	Medical male circumcision	HC	Infectious Disease, Health Services	No	No	Preventive
HC26	For PLHIV and children under five who are close contacts or household members of individuals with active TB, perform symptom screening and chest radiograph; if there is no active TB, provide isoniazid preventive therapy according to current WHO guidelines	HC	Infectious Disease	No	No	Curative
HC27	Diagnosis of TB and first-line treatment	HC	Infectious Disease	No	No	Curative
HC28	Screening for HIV in all individuals with a diagnosis of active TB; if HIV infection is present, start (or refer for) ARV treatment and HIV care	CL	Infectious Disease	No	No	Curative
HC29	Latent-TB screening and IPT for PLHIV	HC	Infectious Disease	No	No	Curative
HC3	Management of premature rupture of membranes, including administration of antibiotics	FLH	RMNCH	Yes	No	Curative
HC30	Fever management for clinically stable	HC	Infectious Disease	No	No	Curative
HC32	Provision of insecticide nets to U5 children and pregnant women attending health centres	HC	Infectious Disease	No	No	Preventive
HC33	Identify and refer for progressive illness **	HC	Infectious Disease	No	No	Curative
HC36	Long-term combination therapy for persons with multiple CVD risk factors, including screening for CVD in community setting using non-lab-based tools to assess overall CVD risk	HC	NCD & IPC	No	No	Curative
HC37	Low-dose inhaled corticosteroids and bronchodilators for asthma and for selected patients with COPD	HC	NCD & IPC	No	No	Curative
HC38	Provision of aspirin for all cases of suspected acute myocardial infarction	HC	NCD & IPC	No	Yes	Curative
HC39a	Screening and ACEi or ARBs for kidney disease	HC	NCD & IPC	No	No	Curative
HC41	Secondary prophylaxis for rheumatic fever	HC	NCD & IPC	No	No	Preventive

DCP3 Code	Intervention name	Platform	Cluster	Vulnerable Population	Rule of rescue	Intervention Purpose
HC42	Treatment of acute pharyngitis for rheumatic fever	HC	NCD & IPC	No	Yes	Curative
HC45	Opportunistic screening for hypertension	HC	NCD & IPC	No	No	Curative
HC46	Tobacco cessation counselling	HC	NCD & IPC	No	No	Promotive
HC48	Support for caregivers of dementia patients	HC	NCD & IPC	No	No	Promotive
HC49	Bipolar disorder management	HC	NCD & IPC	No	No	Curative
HC4a	Provision of condoms and hormonal contraceptives, including emergency contraceptives	CL	RMNCH	Yes	No	Promotive
HC4b	Provision of condoms and hormonal contraceptives, including insertion and removal of contraceptives (PHC)	HC	RMNCH	Yes	No	Promotive
HC50	Management of depression and anxiety disorders with psychological and generic antidepressants therapy	HC	NCD & IPC	No	No	Curative
HC53	Screening and brief alcohol intervention	HC	NCD & IPC	No	No	Preventive
HC55	Primary prevention of osteoporosis	HC	NCD & IPC	No	No	Preventive
HC56	Screening for congenital hearing loss	HC	NCD & IPC	No	No	Curative
HC57a	Dental extraction (PHC)	HC	Health Services	No	No	Curative
HC57b	Dental extraction (FLH)	FLH	Health Services	No	No	Curative
HC58a	Drainage of dental abscess (PHC)	HC	Health Services	No	No	Curative
HC59	Drainage of superficial abscess	HC	Health Services	No	No	Curative
HC5a	Counselling on kangaroo care for new-borns (CL)	CL	RMNCH	Yes	No	Preventive
HC5b	Counselling on kangaroo care for new-borns (PHC)	HC	RMNCH	Yes	No	Preventive
HC6	Management of neonatal sepsis, pneumonia and meningitis using injectable and oral antibiotics	FLH	RMNCH	Yes	No	Curative
HC60	Non-displaced fractures management	HC	Health Services	No	No	Curative
HC61	Resuscitation with basic life support measures	HC	Health Services	No	No	Curative
HC62	Suturing laceration	HC	Health Services	No	No	Curative
HC63a	Treatment of caries (PHC)	HC	Health Services	No	No	Curative
HC64	Basic management of MNIs and disorders	HC	Health Services	No	No	Curative
HC66	Psychosocial support and counselling	CL	NCD & IPC	No	No	Palliative
HC67	Expanded palliative care and pain control measures, including prevention and relief of all physical and psychological symptoms of suffering	FLH	Health Services	No	No	Palliative
HC68	Health centre pathology services **	HC	Health Services	No	No	Curative
HC7	Pharmacological termination of pregnancy	HC	RMNCH	Yes	No	Curative
HC9a	Screening of hypertensive disorders in pregnancy	CL	RMNCH, NCD & IPC	Yes	No	Curative

DCP3 Code	Intervention name	Platform	Cluster	Vulnerable Population	Rule of rescue	Intervention Purpose
HC9b	Screening and management of hypertensive disorders in pregnancy	HC	RMNCH, NCD & IPC	Yes	No	Curative
P5	Systematic identification of individuals with TB symptoms among high-risk groups and linkages to care (active case finding)	CL	Infectious Disease	No	No	Curative
RH1	Full supportive care for preterm new-borns	FLH	RMNCH	Yes	Yes	Curative
RH2	Specialized TB services, including management of MDR- and XDR-TB treatment failure and surgery for TB	RH	Infectious Disease	No	No	Curative
RH3	Management of refractory febrile illness including etiologic diagnosis at reference microbial laboratory	RH	Infectious Disease	No	No	Curative
RH4	Management of acute ventilator failure due to acute exacerbations of asthma and COPD	RH	NCD & IPC	No	Yes	Curative
RH5	Retinopathy screening via telemedicine, followed by treatment using laser photocoagulation	RH	NCD & IPC	No	No	Curative
RH6	Use of percutaneous coronary intervention for acute myocardial infarction where resources permit	RH	NCD & IPC	No	No	Curative
RH7	Treatment of early-stage breast cancer with appropriate multimodal approaches (including generic chemotherapy) with curative intent for cases detected by clinical examination	RH	NCD & IPC	No	No	Curative
RH8	Treatment of early-stage colorectal cancer with appropriate multimodal approaches (including generic chemotherapy) with curative intent for cases detected by clinical examination	RH	NCD & IPC	No	No	Curative
RH9	Treatment of early-stage childhood cancers (such as Burkitt and Hodgkin lymphoma, acute lymphoblastic leukaemia, retinoblastoma and Wilms tumour) with curative intent in paediatric cancer units or hospitals	RH	NCD & IPC	No	No	Curative
RH10	Elective surgical repair of common orthopaedic injuries (for example meniscal and ligamentous tears) in individuals with severe functional limitation	RH	NCD & IPC	No	No	Curative
RH11	Urgent, definitive surgical management of orthopaedic injuries (for example open reduction and internal fixation)	RH	NCD & IPC	No	Yes	Curative
RH12	Repair of cleft lip and cleft palate	RH	NCD & IPC, Health Services	No	No	Curative
RH13	Repair of club foot	RH	NCD & IPC, Health Services	No	No	Curative
RH14	Cataract extraction	FLH	Health Services	No	No	Curative
RH15	Repair of anorectal malformations and Hirschsprung’s disease	RH	Health Services	No	No	Curative
RH16	Repair of obstetric fistula	RH	Health Services	No	Yes	Curative
RH17	Ventriculoperitoneal Shunt	RH	Health Services	No	Yes	Curative
RH18	Surgery for Trachomatous Trichiasis	RH	Health Services	No	No	Curative
RH19	Referral level hospital pathology services	RH	Health Services	No	No	Curative
RH20	Speciality pathology services	RH	Health Services	No	No	Curative
