# Supplementary material for: The Use of Evidence to Design an Essential Package of Health Services in Pakistan: A Review and Analysis of Prioritisation Decisions at Different Stages of the Appraisal Process
Source: Int J Health Policy Manag. 2024 Mar 9;13:8043. doi: 10.34172/ijhpm.2024.8043 (PMC11608344; doi:10.34172/ijhpm.2024.8043)
Supplement: Supplementary file 4 — Supplementary file 3. Status of Intervention Per Stage in the Deliberation Process. [file ijhpm-13-8043-s004.pdf]

Supplementary Information

Article title: The use of evidence to design an essential package of health services in Pakistan: a review and analysis of prioritisation decisions at different stages of the appraisal process

Journal name: International Journal of Health Policy and Management (IJHPM)

Authors' information: Sergio Torres-Rueda[1], Anna Vassall[1]*, Raza Zaidi[2], Nichola Kitson[1], Muhammad Khalid[2], Wahaj Zulfiqar[2], Maarten Jansen[3], Wajeeha Raza[4], Maryam Huda[5], Frank Sandmann[6], Rob Baltussen[3], Sameen Siddiqi[5], Ala Alwan[7]

1. Department of Global Health & Development, London School of Hygiene and Tropical Medicine, London, UK

2. Ministry of National Health Services, Regulations and Coordination, Islamabad, Pakistan

3. Department of Health Evidence, Radboud Institute of Health Sciences, Radboud University Medical Center, Nijmegen, The Netherlands

4. Centre for Health Economics, University of York, York, UK

5. Department of Community Health Sciences, Aga Khan University, Karachi, Pakistan

6. Department of Infectious Disease Epidemiology, London School of Hygiene and Tropical Medicine, London, UK

7. DCP3 Country Translation Project, London School of Hygiene and Tropical Medicine, London, UK

* Corresponding author: anna.vassall@lshtm.ac.uk

Supplementary File 3

Supplementary Table 3: Status of intervention per stage in the deliberation process

Abbreviations: DCP, Disease Control Priorities; EUHC, Essential Universal Health Coverage; TWG, technical working group; NAC, National Advisory Council; EPHS, Essential Package of Health Services; IIP, immediate implementation package; World Health Organization; HiB, Haemophilus influenzae type b; BCG, Bacillus Calmette-Guéri; IRS, indoor residual spraying; HPV, human papillomavirus; STIs, sexually transmitted infections; TB, tuberculosis; WASH, water, sanitation and hygiene; G6PD, glucose-6-phosphate dehydrogenase; BEmONC, Basic Emergency Obstetric and Newborn Care; PLHIV, people living with HIV; IMAI, integrated management of adolescent and adult illness; CVD, cardiovascular disease; COPD, chronic obstructive pulmonary disease; ACEi, angiotensin-converting enzyme inhibitor; ARBs, angiotensin receptor blockers; IV, intravenous; CEmONC, Comprehensive Emergency Obstetric and Newborn Care; ART, antiretroviral treatment; NTDs, neglected tropical diseases; ECD, early childhood development; ARV, antiretroviral; IMCI, Integrated management of childhood illness; IUCDs, IDU, intravenous drug user; MSM, men who have sex with men; MDR, multidrug-resistant tuberculosis; XDR, extensively drug-resistant tuberculosis; C, community; HC, health centre; FLH, first-level hospital; RH, referral hosptal.

Note that the inclusion of interventions in the final packages can be found in Alwan et al. (2023) [1].

DCP code	Intervention name	DCP3 EUHC Shortlist	TWG Combined	NAC 2 Full EPHS	NAC 2 IIP
C1	Antenatal and postpartum education on birth spacing	Shortlisted	High priority	Included	Included
C10	Education on handwashing, personal hygiene and safe disposal of children’s stool	Shortlisted	High priority	Included	Included
C11	Pneumococcus vaccination	Shortlisted	High priority	Included	Included
C12	Rotavirus vaccination	Shortlisted	High priority	Included	Included
C14	Vitamin A and zinc for children	Shortlisted	High priority	Included	Excluded
C16	Childhood vaccination series (diphtheria, pertussis, tetanus, polio, BCG, measles, hepatitis B, HiB, rubella)	Shortlisted	Medium or low priority	Included	Included
C17	Indoor residual spraying	Shortlisted	Medium or low priority	Excluded	Excluded
C18	Education of schoolchildren on oral health	Shortlisted	High priority	Included	Excluded
C19	Vision pre-screening by teachers; vision tests and provision of ready-made glasses on-site by eye specialists	Shortlisted	High priority	Included	Included
C2	Counselling of mothers on providing thermal care for pre- term new-borns (delayed bath and skin to skin contact)	Shortlisted	High priority	Included	Included
C20	School based HPV vaccination for girls	Shortlisted	High priority	Excluded	Excluded

DCP code	Intervention name	DCP3 EUHC Shortlist	TWG Combined	NAC 2 Full EPHS	NAC 2 IIP
C21	Mass drug administration (NTDs)	Shortlisted	Medium or low priority	Excluded	Excluded
C23	Adolescent-friendly services for STIs	Shortlisted	High priority	Excluded	Excluded
C24	Life skills training in schools	Shortlisted	High priority	Excluded	Excluded
C27a	Provision of iron and folic acid supplementation to pregnant women, and provision of food or caloric supplementation to pregnant women in food-insecure households	Shortlisted	High priority	Included	Included
C27b	Provision of iron and folic acid supplementation to pregnant women, and provision of food or caloric supplementation to pregnant women in food-insecurity households	Shortlisted	High priority	Included	Included
C28	Community-based HIV testing and counselling (for example, mobile units and venue-based testing), with appropriate referral or linkage to care and	Shortlisted	High priority	Included	Included
C30a	Provision of condoms to key populations, including sex workers, men who have sex with men, people who inject drugs, transgender populations, and prisoners	Shortlisted	High priority	Included	Included
C30b	Provision of Disposable syringes who inject drugs (IDU)	Shortlisted	High priority	Included	Included
C32	Routine contact tracing to identify individuals exposed to TB and link them to care	Shortlisted	High priority	Included	Included
C33	Test for G6PD deficiency	Shortlisted	Medium or low priority	Included	Excluded
C3a	Management of labour and delivery in low-risk women by skilled attendant (CL)	Shortlisted	High priority	Included	Included
C3b	Basic neonatal resuscitation following delivery (CL)	Shortlisted	High priority	Included	Included
C3c	Management of labour and delivery in low-risk women by skilled attendant (PHC)	Shortlisted	High priority	Included	Included
C3d	Basic neonatal resuscitation following delivery (PHC)	Shortlisted	High priority	Included	Included
C34	Environmental management for malaria	Shortlisted	High priority	Excluded	Excluded
C4	Promotion of breastfeeding and complementary feeding by community health workers	Shortlisted	High priority	Included	Included
C41	Mass drug administration (malaria)	Shortlisted	Medium or low priority	Excluded	Excluded
C43	Early detection and treatment of leishmaniasis, dengue, chikungunya, rabies, trachoma and helminthiasis.	Shortlisted	Medium or low priority	Included	Included
C45	Identify and refer patients with high risk	Shortlisted	High priority	Included	Excluded
C46	In the context of an emerging infectious outbreak, provide advice and guidance on how to recognize early symptoms and signs and when to seek medical attention	Shortlisted	High priority	Included	Excluded
C47	Exercise-based pulmonary rehabilitation	Shortlisted	Medium or low priority	Excluded	Excluded

DCP code	Intervention name	DCP3 EUHC Shortlist	TWG Combined	NAC 2 Full EPHS	NAC 2 IIP
C48	Self-managed treatment of migraine	Shortlisted	Medium or low priority	Excluded	Excluded
C5	Tetanus toxoid immunization among schoolchildren and women attending antenatal care	Shortlisted	High priority	Included	Included
C50	Parent training of high-risk families, including nurse home visitation for child maltreatment	Shortlisted	High priority	Excluded	Excluded
C51	WASH behaviour change interventions, such as community led total sanitation	Shortlisted	High priority	Included	Included
C53a	Identification/screening of the early childhood development issues motor, sensory and language stimulation	Shortlisted	High priority	Included	Excluded
C53b	ECD rehabilitation interventions	Shortlisted	High priority	Included	Excluded
C56	Interventions for wheelchair users	Shortlisted	Medium or low priority	Excluded	Excluded
C8	Acute severe malnutrition management	Shortlisted	High priority	Included	Excluded
C9	Integrated community case management	Shortlisted	Medium or low priority	Excluded	Excluded
FLH1	Care for foetal growth restriction	Shortlisted	High priority	Included	Excluded
FLH10	Surgical termination of pregnancy by maternal vacuum aspiration and dilatation & curettage	Shortlisted	Medium or low priority	Included	Included
FLH11	Care for severe childhood infections	Shortlisted	High priority	Included	Excluded
FLH12	Severe acute malnutrition management	Shortlisted	High priority	Included	Excluded
FLH13	Early detection and treatment of early-stage cervical cancer	Shortlisted	High priority	Included	Included
FLH14	Insertion and removal of contraceptives	Shortlisted	High priority	Included	Excluded
FLH15	Tubal ligation	Shortlisted	High priority	Included	Excluded
FLH16	Vasectomy	Shortlisted	High priority	Included	Excluded
FLH17	Referral of cases of treatment failure for drug susceptibility testing; enrolment of those with MDR-TB for treatment per WHO guidelines (either short- or long-term regimen)	Shortlisted	High priority	Included	Included
FLH18	Evaluation and management of fever in clinically unstable individuals using WHO IMAI guidelines, including empiric parenteral antimicrobials and antimalarial and resuscitative measures for septic shock	Shortlisted	High priority	Included	Included
FLH2	Induction of labour post-term	Shortlisted	Medium or low priority	Excluded	Excluded
FLH20	Management of acute coronary syndromes	Shortlisted	Medium or low priority	Included	Excluded
FLH22	Management of acute exacerbations of asthma and COPD using systemic steroids, inhaled beta-agonists and if indicated oral antibiotics and oxygen therapy	Shortlisted	High priority	Included	Included
FLH23	Medical management of acute heart failure	Shortlisted	High priority	Included	Included

DCP code	Intervention name	DCP3 EUHC Shortlist	TWG Combined	NAC 2 Full EPHS	NAC 2 IIP
FLH24	Bowel obstruction management	Shortlisted	High priority	Included	Included
FLH25	Calcium and vitamin D supplementation for secondary prevention of osteoporosisE264	Shortlisted	High priority	Excluded	Excluded
FLH26	Combination therapy, including low-dose corticosteroids and generic disease-modifying antirheumatic drugs (including methotrexate), for individuals with moderate to severe rheumatoid arthritis	Shortlisted	Medium or low priority	Excluded	Excluded
FLH27	In settings where sickle cell disease is a public health concern, universal newborn screening followed by standard prophylaxis against bacterial infections and malaria	Shortlisted	Medium or low priority	Excluded	Excluded
FLH28	In setting where specific single-gene disorders are a public health concern (for example thalassemia), retrospective identification of carriers plus prospective (premarital) screening and counselling to reduce rates of conception	Shortlisted	Medium or low priority	Excluded	Excluded
FLH3	Jaundice management with phototherapy	Shortlisted	High priority	Included	Included
FLH30	Intoxication/poisoning management	Shortlisted	High priority	Included	Excluded
FLH31	Appendectomy	Shortlisted	High priority	Included	Excluded
FLH32	Assisted vaginal delivery using vacuum extraction or forceps	Shortlisted	Medium or low priority	Excluded	Excluded
FLH33	Craniotomy for trauma	Shortlisted	Medium or low priority	Excluded	Excluded
FLH34	Colostomy for acute bowel obstruction/volvulus and injuries.	Shortlisted	High priority	Included	Included
FLH35	Escharotomy or fasciotomy	Shortlisted	High priority	Included	Included
FLH36	Management of non-displaced fractures	Shortlisted	High priority	Included	Included
FLH37	Hernia Repair	Shortlisted	Medium or low priority	Excluded	Excluded
FLH38	Hysterectomy for uterine rupture or intractable postpartum haemorrhage	Shortlisted	High priority	Included	Included
FLH39	Irrigation and debridement of open fractures	Shortlisted	High priority	Included	Included
FLH4	Eclampsia management with magnesium sulphate, including initial stabilization at health centres	Shortlisted	High priority	Included	Included
FLH40	Management of osteomyelitis, including surgical debridement	Shortlisted	Medium or low priority	Excluded	Excluded
FLH41a	Management of Septic Arthritis	Shortlisted	High priority	Included	Included
FLH41b	Placement of External Fixation and Use of Traction for Fractures	Shortlisted	High priority	Included	Included
FLH42	Relief of urinary obstruction by catheterization for fractures	Shortlisted	High priority	Included	Included
FLH43	Removal of gallbladder, including emergency surgery	Shortlisted	High priority	Included	Included
FLH44	Repair of perforations (for example perforated peptic ulcer, typhoid ileal perforation)	Shortlisted	High priority	Included	Included

DCP code	Intervention name	DCP3 EUHC Shortlist	TWG Combined	NAC 2 Full EPHS	NAC 2 IIP
FLH45	Resuscitation with advanced measures	Shortlisted	High priority	Included	Excluded
FLH46	Basic Skin grafting	Shortlisted	Medium or low priority	Excluded	Excluded
FLH48a	Trauma laparotomy	Shortlisted	High priority	Included	Included
FLH49	Trauma-related amputations	Shortlisted	High priority	Included	Included
FLH5	Maternal sepsis management	Shortlisted	High priority	Included	Excluded
FLH50	Tube thoracostomy	Shortlisted	High priority	Included	Excluded
FLH52	Compression therapy for amputations, burns, and vascular or lymphatic disorders	Shortlisted	High priority	Included	Included
FLH53	Evaluation and acute management of swallowing dysfunctionE307	Shortlisted	Medium or low priority	Excluded	Excluded
FLH57	Prevention and relief of refractory suffering and acute pain related to surgery, serious injury or other serious, complex or life-limiting health problems	Shortlisted	High priority	Excluded	Excluded
FLH58	First level hospital pathology services	Shortlisted	High priority	Excluded	Excluded
FLH6	Management of new-born complications infections, meningitis, septicaemia, pneumonia and other very serious infections requiring continuous supportive care (such as IV fluids and oxygen)	Shortlisted	High priority	Included	Included
FLH7	Preterm labour management	Shortlisted	High priority	Included	Excluded
FLH8	Management of labour and delivery in high-risk women, including operative delivery (CEmONC)	Shortlisted	Medium or low priority	Included	Excluded
FLH9	Surgery for ectopic pregnancy	Shortlisted	Medium or low priority	Excluded	Excluded
HC1	Early detection and treatment of neonatal pneumonia with oral antibiotics	Shortlisted	High priority	Included	Included
HC10	Screening and management of diabetes (gestational diabetes or pre-existing type II diabetes)	Shortlisted	Medium or low priority	Included	Included
HC11	Management of labour and delivery in low-risk women (BEmONC), including initial treatment of obstetric or delivery complications prior to transfer (Also included in Surgery package of services)	Shortlisted	High priority	Included	Included
HC12	Detection and treatment of childhood infections with danger signs (IMCI)	Shortlisted	High priority	Included	Included
HC13	Among all individuals who are known to be HIV+, immediate ART initiation with regular monitoring of viral load for	Shortlisted	High priority	Excluded	Excluded
HC14	Psychological treatment	Shortlisted	High priority	Included	Excluded
HC16	Post gender-based violence care	Shortlisted	High priority	Included	Excluded
HC17	Syndromic management of common sexual and reproductive tract infections (for example urethral discharge, genital ulcer and others)	Shortlisted	High priority	Included	Included

DCP code	Intervention name	DCP3 EUHC Shortlist	TWG Combined	NAC 2 Full EPHS	NAC 2 IIP
HC19	For individuals testing positive for hepatitis B and C, assessment of treatment eligibility by trained providers followed by initiation and monitoring of ART when indicated	Shortlisted	High priority	Included	Included
HC2	Miscarriage and abortions management	Shortlisted	Medium or low priority	Included	Excluded
HC20	Hepatitis B and C testing of high-risk individuals identified in the national testing policy with appropriate referral of positive individuals to trained providers	Shortlisted	High priority	Included	Included
HC21	Partner notification and expedited treatment for common STIs including HIV	Shortlisted	High priority	Included	Included
HC23	Provider-initiated testing and counselling for HIV, STIs and hepatitis for all in contact with the health system in high- prevalence setting, including prenatal care with appropriate referral/ linkages to care including immediate ART initiation for those testing positive for HIV	Shortlisted	High priority	Included	Included
HC24	Hepatitis B vaccination for high-risk populations, including healthcare workers, IDU, MSM, household contacts and partners with multiple sex partners	Shortlisted	High priority	Included	Included
HC25	Medical male circumcision	Shortlisted	High priority	Included	Excluded
HC26	For PLHIV and children under five who are close contacts or household members of individuals with active TB, perform symptom screening and chest radiograph; if there is no active TB, provide isoniazid preventive therapy according to current WHO guidelines	Shortlisted	High priority	Included	Included
HC27	Diagnosis of TB and first-line treatment	Shortlisted	High priority	Included	Included
HC28	Screening for HIV in all individuals with a diagnosis of active TB; if HIV infection is present, start (or refer for) ARV treatment and HIV care	Shortlisted	High priority	Included	Included
HC29	Latent-TB screening and IPT for PLHIV	Shortlisted	Medium or low priority	Excluded	Excluded
HC3	Management of premature rupture of membranes, including administration of antibiotics	Shortlisted	Medium or low priority	Included	Included
HC30	Fever management for clinically stable	Shortlisted	Medium or low priority	Included	Excluded
HC32	Provision of insecticide nets to U5 children and pregnant women attending health centres	Shortlisted	Medium or low priority	Included	Included
HC33	Identify and refer for progressive illness **	Shortlisted	High priority	Included	Excluded
HC36	Long-term combination therapy for persons with multiple CVD risk factors, including screening for CVD in community setting using non-lab-based tools to assess overall CVD risk	Shortlisted	High priority	Included	Included
HC37	Low-dose inhaled corticosteroids and bronchodilators for asthma and for selected patients with COPD	Shortlisted	High priority	Included	Included
HC38	Provision of aspirin for all cases of suspected acute myocardial infarction	Shortlisted	High priority	Included	Included

DCP code	Intervention name	DCP3 EUHC Shortlist	TWG Combined	NAC 2 Full EPHS	NAC 2 IIP
HC39a	Screening and ACEi or ARBs for kidney disease	Shortlisted	High priority	Included	Excluded
HC41	Secondary prophylaxis for rheumatic fever	Shortlisted	Medium or low priority	Included	Excluded
HC42	Treatment of acute pharyngitis for rheumatic fever	Shortlisted	High priority	Included	Included
HC45	Opportunistic screening for hypertension	Shortlisted	High priority	Included	Excluded
HC46	Tobacco cessation counselling	Shortlisted	High priority	Excluded	Excluded
HC48	Support for caregivers of dementia patients	Shortlisted	Medium or low priority	Excluded	Excluded
HC49	Bipolar disorder management	Shortlisted	Medium or low priority	Excluded	Excluded
HC4a	Provision of condoms and hormonal contraceptives, including emergency contraceptives	Shortlisted	High priority	Included	Included
HC4b	Provision of condoms and hormonal contraceptives, including insertion and removal of contraceptives (PHC)	Shortlisted	High priority	Included	Included
HC50	Management of depression and anxiety disorders with psychological and generic antidepressants therapy	Shortlisted	High priority	Included	Included
HC53	Screening and brief alcohol intervention	Shortlisted	High priority	Excluded	Excluded
HC55	Primary prevention of osteoporosis	Shortlisted	High priority	Excluded	Excluded
HC56	Screening for congenital hearing loss	Shortlisted	Medium or low priority	Included	Excluded
HC57a	Dental extraction (PHC)	Shortlisted	High priority	Included	Excluded
HC57b	Dental extraction (FLH)	Shortlisted	High priority	Included	Excluded
HC58a	Drainage of dental abscess (PHC)	Shortlisted	High priority	Included	Excluded
HC59	Drainage of superficial abscess	Shortlisted	High priority	Included	Excluded
HC5a	Counselling on kangaroo care for new-borns (CL)	Shortlisted	High priority	Included	Included
HC5b	Counselling on kangaroo care for new-borns (PHC)	Shortlisted	High priority	Included	Included
HC6	Management of neonatal sepsis, pneumonia and meningitis using injectable and oral antibiotics	Shortlisted	High priority	Included	Included
HC60	Non-displaced fractures management	Shortlisted	Medium or low priority	Included	Excluded
HC61	Resuscitation with basic life support measures	Shortlisted	High priority	Included	Included
HC62	Suturing laceration	Shortlisted	High priority	Included	Included
HC63a	Treatment of caries (PHC)	Shortlisted	Medium or low priority	Included	Included
HC64	Basic management of MNIs and disorders	Shortlisted	High priority	Included	Excluded

DCP code	Intervention name	DCP3 EUHC Shortlist	TWG Combined	NAC 2 Full EPHS	NAC 2 IIP
HC66	Psychosocial support and counselling	Shortlisted	Medium or low priority	Excluded	Excluded
HC67	Expanded palliative care and pain control measures, including prevention and relief of all physical and psychological symptoms of suffering	Shortlisted	High priority	Excluded	Excluded
HC68	Health centre pathology services **	Shortlisted	High priority	Included	Excluded
HC7	Pharmacological termination of pregnancy	Shortlisted	Medium or low priority	Included	Included
HC9a	Screening of hypertensive disorders in pregnancy	Shortlisted	High priority	Included	Included
HC9b	Screening and management of hypertensive disorders in pregnancy	Shortlisted	High priority	Included	Included
P5	Systematic identification of individuals with TB symptoms among high-risk groups and linkages to care (active case finding)	Shortlisted	High priority	Included	Excluded
RH1	Full supportive care for preterm new-borns	Shortlisted	High priority	Included	Included
RH2	Specialized TB services, including management of MDR- and XDR-TB treatment failure and surgery for TB	Shortlisted	High priority	Excluded	Excluded
RH3	Management of refractory febrile illness including etiologic diagnosis at reference microbial laboratory	Shortlisted	High priority	Excluded	Excluded
RH4	Management of acute ventilator failure due to acute exacerbations of asthma and COPD	Shortlisted	High priority	Excluded	Excluded
RH5	Retinopathy screening via telemedicine, followed by treatment using laser photocoagulation	Shortlisted	High priority	Excluded	Excluded
RH6	Use of percutaneous coronary intervention for acute myocardial infarction where resources permit	Shortlisted	High priority	Excluded	Excluded
RH7	Treatment of early-stage breast cancer with appropriate multimodal approaches (including generic chemotherapy) with curative intent for cases detected by clinical examination	Shortlisted	High priority	Excluded	Excluded
RH8	Treatment of early-stage colorectal cancer with appropriate multimodal approaches (including generic chemotherapy) with curative intent for cases detected by clinical examination	Shortlisted	High priority	Excluded	Excluded
RH9	Treatment of early-stage childhood cancers (such as Burkitt and Hodgkin lymphoma, acute lymphoblastic leukaemia, retinoblastoma and Wilms tumour) with curative intent in paediatric cancer units or hospitals	Shortlisted	Medium or low priority	Excluded	Excluded
RH10	Elective surgical repair of common orthopaedic injuries (for example meniscal and ligamentous tears) in individuals with severe functional limitation	Shortlisted	Medium or low priority	Excluded	Excluded
RH11	Urgent, definitive surgical management of orthopaedic injuries (for example open reduction and internal fixation)	Shortlisted	Medium or low priority	Excluded	Excluded
RH12	Repair of cleft lip and cleft palate	Shortlisted	High priority	Excluded	Excluded
RH13	Repair of club foot	Shortlisted	High priority	Excluded	Excluded
RH14	Cataract extraction	Shortlisted	High priority	Included	Excluded

DCP code	Intervention name	DCP3 EUHC Shortlist	TWG Combined	NAC 2 Full EPHS	NAC 2 IIP
RH15	Repair of anorectal malformations and Hirschsprung's disease	Shortlisted	High priority	Excluded	Excluded
RH16	Repair of obstetric fistula	Shortlisted	High priority	Excluded	Excluded
RH17	Ventriculoperitoneal Shunt	Shortlisted	High priority	Excluded	Excluded
RH18	Surgery for Trachomatous Trichiasis	Shortlisted	High priority	Excluded	Excluded
RH19	Referral level hospital pathology services	Shortlisted	High priority	Excluded	Excluded
RH20	Speciality pathology services	Shortlisted	High priority	Excluded	Excluded

References

1. Alwan A, Siddiqi S, Safi M, et al. Addressing the UHC challenge using the Disease Control Priorities 3 approach: lessons learned and an overview of the Pakistan experience. Int J Health Policy Manag. 2023;12:8003. doi:10.34172/ijhpm.2023.8003
